# Increased PKC activity and altered GSK3β/NMDAR function drive behavior cycling in HINT1-deficient mice: bipolarity or opposing forces

**DOI:** 10.1038/srep43468

**Published:** 2017-02-27

**Authors:** Javier Garzón-Niño, María Rodríguez-Muñoz, Elsa Cortés-Montero, Pilar Sánchez-Blázquez

**Affiliations:** 1 Neuropharmacology, Department of Translational Neurosciences, Instituto Cajal, CSIC, Madrid E-28002, Spain

## Abstract

Mice with histidine triad nucleotide-binding protein 1 (HINT1) deletion exhibit manic-like symptoms that evolve into depressive-like behavior in response to stressful paradigms. Molecular and electrophysiological studies have indicated that HINT1^−/−^ mice exhibit increased PKC, PKA, and GSK3β activities, as well as glutamate *N*-methyl-D-aspartate receptor (NMDAR)/α-amino-3-hydroxy-5-methyl-4-isoxazolepropionic receptor (AMPAR) and NR2B/NR2A subunit ratios. Pharmacological interventions stabilized their behavior but through different mechanisms. GSK3β inhibitors and valproate directly attenuated the expression of the manic-like symptoms, whereas PKC inhibition, lamotrigine, or risperidone promoted NMDAR-mediated depressive-like behaviors that counterbalanced the preexisting manic-like symptoms. Naïve HINT1^−/−^ mice exposed to stressful paradigms rapidly manifested depressive-like behaviors in subsequent stressful situations, a capacity that persisted for a couple of weeks thereafter. During the depressive-like phase, citalopram, amitriptyline and MK801 precipitated manic-like behaviors in stressed HINT1^−/−^ mice. Notably, the antagonism of NMDARs prevented HINT1^−/−^ mice from alternating behaviors in response to stress. A comparison with “manic” Black Swiss mice indicated that in HINT1^−/−^ mice, PKC supports manic-like symptoms and reduces the expression of depressive-like behaviors via activation of GSK3β and regulation of NR2B-enriched NMDARs. HINT1^−/−^ mice represent a suitable model for studying human BPD and may facilitate the identification of novel targets and drugs to treat this mental disorder.

Human bipolar disorder (BPD) is a chronic and debilitating illness with alternating periods of mania, *i.e*., impulsivity, increased energy and hyperactivity, reduced anxiety, and decreased need for sleep, and depression, *i.e*., helplessness, reduced energy and activity, and anhedonia^1^. It has been proposed that manic/hypomanic episodes manifest in response to goal attainment events, circadian rhythm disruptions, spring/summer seasonal conditions, recreational stimulant use, and antidepressant medications, and that depressive episodes develop mainly because of stressful life events, general stress, fatigue, sleep deprivation, physical injury, or illness^2^,^3^,^4^.

Several currently available animal models reproduce select behavioral facets of human mania and depression and can be used to reliably detect novel drugs that may be administered for palliative treatment of these diseases. However, in the case of BPD, there is no single valid and comprehensive animal model for reproducing the fluctuating moods of affected patients^5^. To circumvent this issue, researches have devised strategies that take advantage of the differences between mouse strains to perform coherent and comprehensive batteries of behavioral tests. Among others putative models^1^,^6^,^7^, flinders sensitive rat lines (FSLs), which exhibit depression-like behaviors^8^, and Black Swiss (BS) mice from Taconic Farms (BStac), which exhibit manic-like behaviors^9^, are currently being utilized to study the depressive and manic aspects of BPD.

Thus, the availability of an animal exhibiting the relevant behavioral features of this complex human disease would assist in the development of new drugs for treating BPD. If molecular alterations such as those described for BPD patients were also present in this animal, the animal would be a suitable model for studying molecular aspects of human BPD. Thus, mice with a genetic deletion of histidine triad nucleotide-binding protein 1 (HINT1) drew our attention as a reliable surrogate animal model of BPD. Human BPD has consistently been linked to concurrent abnormalities in protein kinase C (PKC) activity and glutamatergic transmission^10^,^11^,^12^,^13^. Enhanced PKC function^14^ and, to a lesser extent, enhanced PKA function^15^ are associated with BPD manic episodes. Elevated glutamate *N*-methyl-D-aspartate (NMDA) receptor signaling, which is commonly observed in patients suffering from depression, has also been noted in patients with bipolar depression^16^,^17^. HINT1^−/−^ mice spontaneously exhibit increased PKC^18^,^19^ and PKA^20^ activity in conjunction with abnormal NMDA receptor (NMDAR) function^20^. The HINT1 protein comprises 126 amino acids, weighs approximately 14 kDa, and belongs to the histidine triad (HIT) family. HINT1 is widely expressed in the CNS^21^ and interacts with sigma 1 receptors in the cell plasma membrane to coordinate G-protein coupled receptor (GPCR) and NMDAR activity^22^. HINT1 binds zinc ions to inhibit conventional PKCγ/PKCα isoenzymes and also interacts with Raf-1^23^. In the absence of HINT1, PKC activity increases, cross-regulation between GPCRs and NMDARs diminishes, and NMDAR responsiveness to direct activation increases^20^.

The *HINT1* gene has been related to BPD^24^,^25^. HINT1^−/−^ mice exhibit an increased activity^26^ and are more responsive to motor stimulation by amphetamines than HINT1^+/+^ wild-type (WT) mice^27^. In the present study, we compared the behaviors and molecular features of HINT1^−/−^ mice with those of BStac mice, an animal model for manic-like behaviors, and compared the responses of these parameters to a series of pharmacological interventions. HINT1^−/−^ and BStac mice displayed manic-like behaviors that remitted in response to mood stabilizers but were insensitive to drugs with antidepressant activity in humans. In HINT1^−/−^ mice, but not in BStac mice, emotional stress precipitated antidepressant-sensitive states that could also be managed with NMDAR blockers. PKC inhibition reduced manic-like symptoms in HINT1^−/−^ mice but exerted only limited effects in HINT1^+/+^ WT and BStac mice. Therefore, HINT1^−/−^ mice exhibit certain behavioral and molecular features of human BPD, and PKC and NMDARs contribute to the expression of this abnormal phenotype.

## Results

### Characterization of HINT1^−/−^ mice

**Figure 1:**
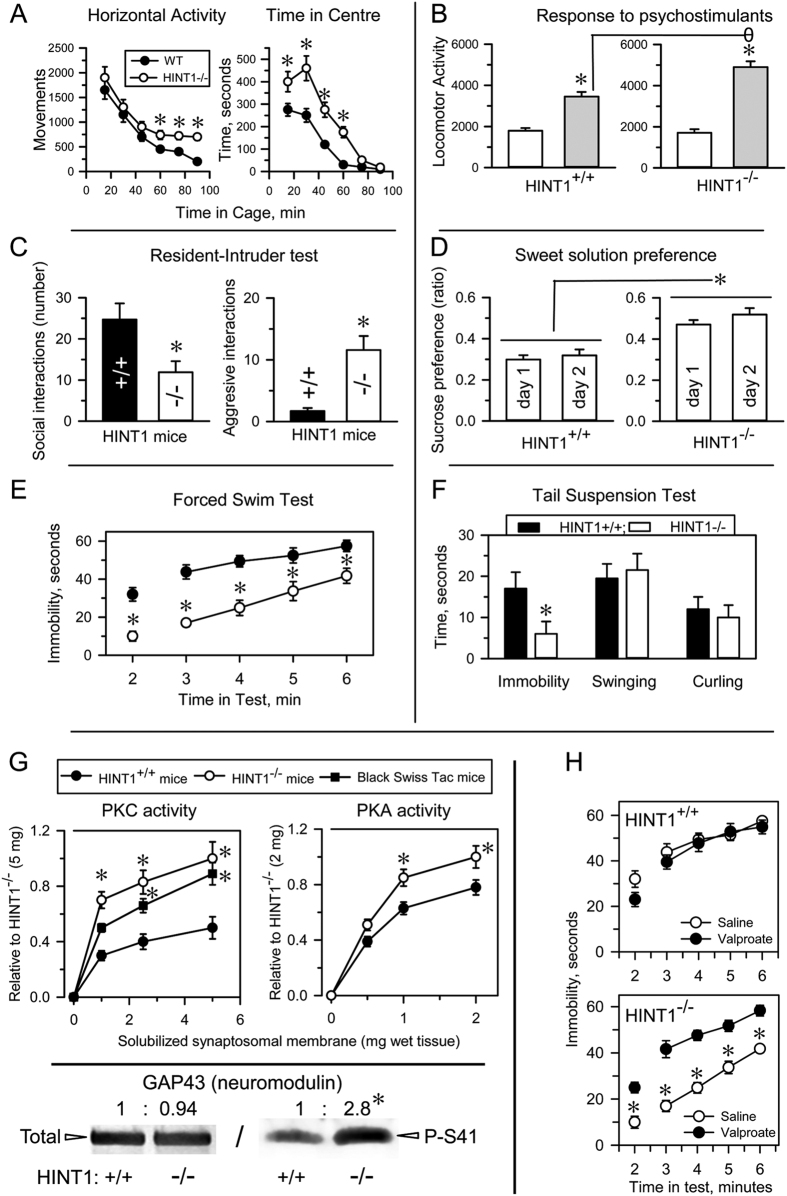
HINT1^−/−^ mice exhibit manic-like behaviors. (**A**) Locomotor habituation and time on center (risk taking behavior) exhibited by HINT1^+/+^ WT and HINT1^−/−^ male mice; (**B**) response to acute systemic saline and apomorphine (5 mg/kg, ip) administration (hypersensitivity to psychostimulants); (**C**) social and aggressive interactions; (**D**) sweet solution preference ratios (hedonistic drive) on days 1 and 2 of the test; (**E,F**) immobility time (goal-directed activity) in the forced swim test (FST) and tail suspension test (TST). Each assay was performed on different cohorts of mice. The results are expressed as the mean ± SEM of total scores (n = 12–15/group). (**G**) PKC and PKA enzymatic activity in frontal cortices from HINT1^+/+^ WT, HINT1^−/−^, and BStac mice. *Significantly different from the HINT1^+/+^ group; ANOVA, all pairwise Holm-Sidak multiple comparison tests, *p* < 0.05. Lower panel: PKC substrate GAP43 and its phosphorylation at serine41 (P-S41) in frontal cortices obtained from HINT1^+/+^ WT and HINT1^−/−^ mice. Immunosignals (average optical density of the pixels within the object area/mm^2^, Quantity One Software, Bio-Rad, Madrid, Spain) were expressed as changes relative to the HINT1^+/+^ WT group (assigned an arbitrary value of 1). The assay was repeated three times, and tubulin was used as a loading control. *Significantly different from HINT1^+/+^ WT, paired t test, *p* < 0.05. (**H**) Treatment with the mood stabilizer valproate (200 mg/kg, ip) augmented the immobility scores of HINT1^−/−^ mice during the FST without affecting the performances of HINT1^+/+^ WT mice. *Significantly different from the saline-treated group; ANOVA, all pairwise Holm-Sidak multiple comparison tests, *p* < 0.05. Ip (intraperitoneal).

We tested HINT1^−/−^ mice and their HINT1^+/+^ WT littermates in a battery of experimental paradigms representing specific behavioral domains of human mania. This battery included spontaneous activity test, sweet solution preference test, resident-intruder test, tail suspension test (TST), forced swim test (FST) and amphetamine-induced hyperactivity test. HINT1^−/−^ mice remained in the open area of the observation field for longer periods than their counterparts and also exhibited higher motor sensitivity to psychostimulants, higher aggression levels in the resident/intruder test and a greater preference for sweetened solutions than HINT1^+/+^ WT mice. Moreover, HINT1^−/−^ mice displayed higher activity levels than HINT1^+/+^ WT mice during stressful situations, such as the TST or FST (Fig. 1A–F), and PKC and PKA activity levels were higher in HINT1^−/−^ mice than in HINT1^+/+^ WT mice. Accordingly, HINT1^−/−^ mice exhibited increased serine phosphorylation of the PKC substrate neuromodulin (GAP43) (Fig. 1G). Mood stabilizers, such as valproate, and PKC inhibitors (iPKCs) diminished HINT1^−/−^ mice activity levels in the FST and TST, lessened their preference for sweet solutions and weakened the effects of psychostimulant on their activity levels (Fig. 1H and Supp. Fig. 1).

**Figure 2:**
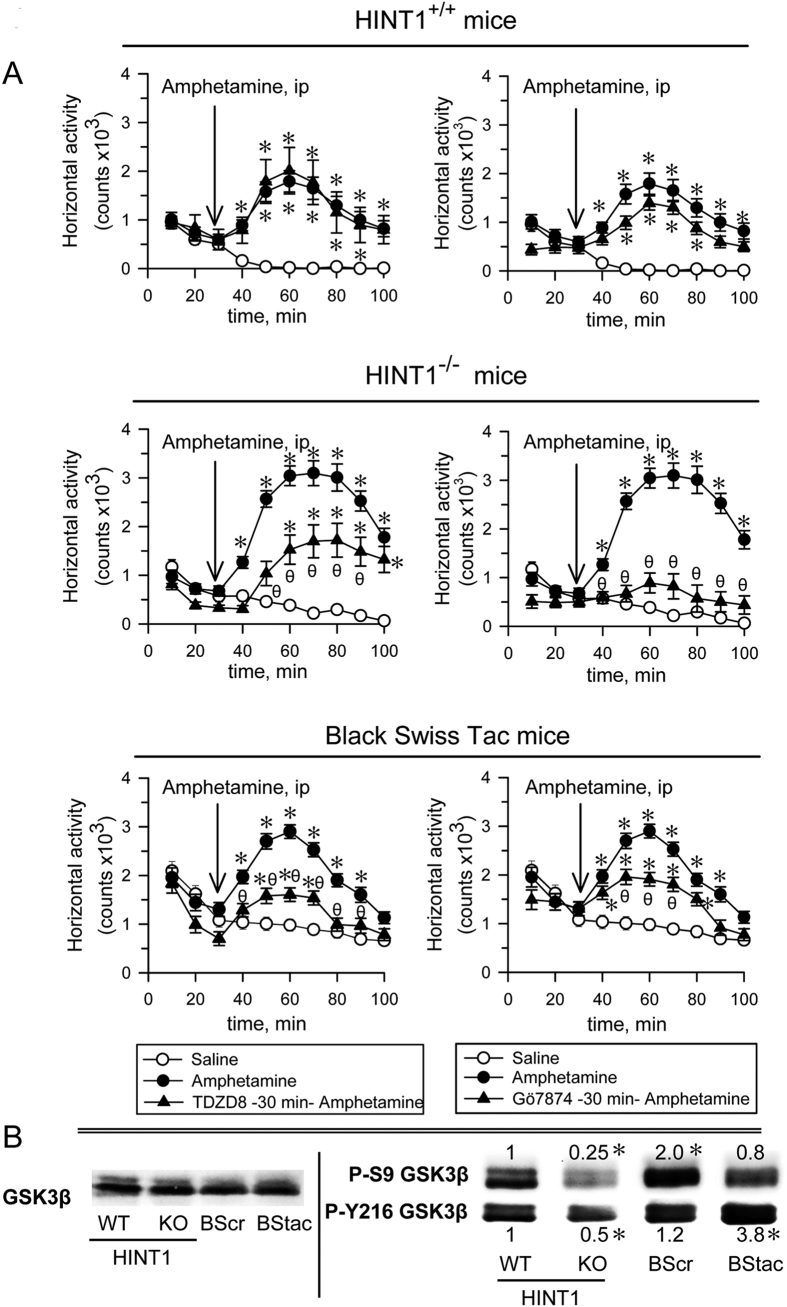
The influence of PKC and GSK3β inhibitors on the hyperactivity evoked by amphetamine in HINT1^+/+^ WT, HINT1^−/−^ and BStac mice. (**A**) Horizontal activity of HINT1^+/+^ WT HINT1^−/−^, and BStac mice pre-treated (at time 0) with vehicle (saline), TDZD8 (20 nmol, icv), or Gö7874 (1 nmol, icv). After 30 min of activity, the mice received saline or amphetamine (2 mg/kg, ip) and were observed during the subsequent 70 min period. Each point is the computed mean ± SEM of the groups. *Significantly different from the control group, which received vehicle instead of amphetamine; θ significantly different from the group that received amphetamine and vehicle instead of the kinase inhibitor. ANOVA, Dunnett multiple comparisons vs control group, *p* < 0.05. (**B**) Total and phosphorylated GSK3β in the synaptosomes of frontal cortices obtained from HINT1^+/+^ WT, HINT1^−/−^ KO, BScr, and BStac mice. Within each row, the P-S9 and Y216 values of the KO, BScr and BStac mice were compared to those of the WT mice (assigned an arbitrary value of 1). *Significantly different from the control group, ANOVA, Dunnett multiple comparisons vs control group, *p* < 0.05. Representative blots are shown (details, see Figure 1). Icv (intracerebroventricular).

Therefore, the behavior of HINT1^−/−^ mice differs from that of HINT1^+/+^ WT mice, as the former exhibits typical manic-like behavior features^28^. We then compared the behavior of HINT1^−/−^ mice with that of BStac mice, an animal model for human mania that also exhibits increased PKC activity levels (Fig. 1G). In the activity cage, BStac mice exhibited a horizontal activity level greater than that of HINT1^−/−^ and HINT1^+/+^ WT mice. Amphetamine elicited significant increases in locomotor activity in HINT1^−/−^ and BStac mice but had less of an effect on HINT1^+/+^ WT mice. It is known that inhibition of GSK3 or of PKC reduces amphetamine-evoked hyperactivity in “manic” BStac mice^29^,^30^. In our assays, inhibition of GSK3β (TDZD8) or PKC (Gö7874) did not attenuate the effects of amphetamine on HINT1^+/+^ WT mice but did reduce the effects of amphetamine on BStac and HINT1^−/−^ mice. PKC inhibition was particularly effective in minimizing the effects of amphetamine on HINT1^−/−^ mice (Fig. 2A). Taken together, these data suggest that targeted *HINT1* gene deletion facilitates the expression of manic-like behaviors.

We then evaluated the presence and regulatory phosphorylations of GSK3β, an enzyme that exhibits alterations in most mood disorders. We included Black Swiss mice from Charles River (BScr) for some parts of the study as a putative control of BStac mice. Although BScr share a genetic background with BStac mice, they do not exhibit the manic-like behavioral phenotype^31^. The molecular exploration showed that total GSK3β levels in the frontal cortex were not significantly different among the four groups of mice. However, phosphorylation analysis revealed marked differences in the regulation of GSK3β. Using HINT1^+/+^ WT mice data as a reference, we observed that Akt-mediated inhibitory phosphorylation of GSK3β at serine 9 (P-S9)^32^ decreased in HINT1^−/−^ mice but increased in BScr mice. Regarding the activating phosphorylation of GSK3β at tyrosine 216 (Y216)^33^, BStac mice exhibited higher P-Y216 GSK3β levels that did HINT1^+/+^ WT mice and BScr mice (Fig. 2B). Manic-like behavior is usually associated with high GSK3β activity and ceases abruptly after GSK3β pharmacological inhibition^34^. BStac and HINT1^−/−^ mice exhibited manic-like behaviors but also showed differences in their cortical levels of the phosphorylated and non-phosphorylated forms of GSK3β. As the activating P-Y216 predominated over the inhibitory P-S9 in both groups of mice, their resulting GSK3β activity could be higher than in HINT1^+/+^ WT mice.

**Figure 3:**
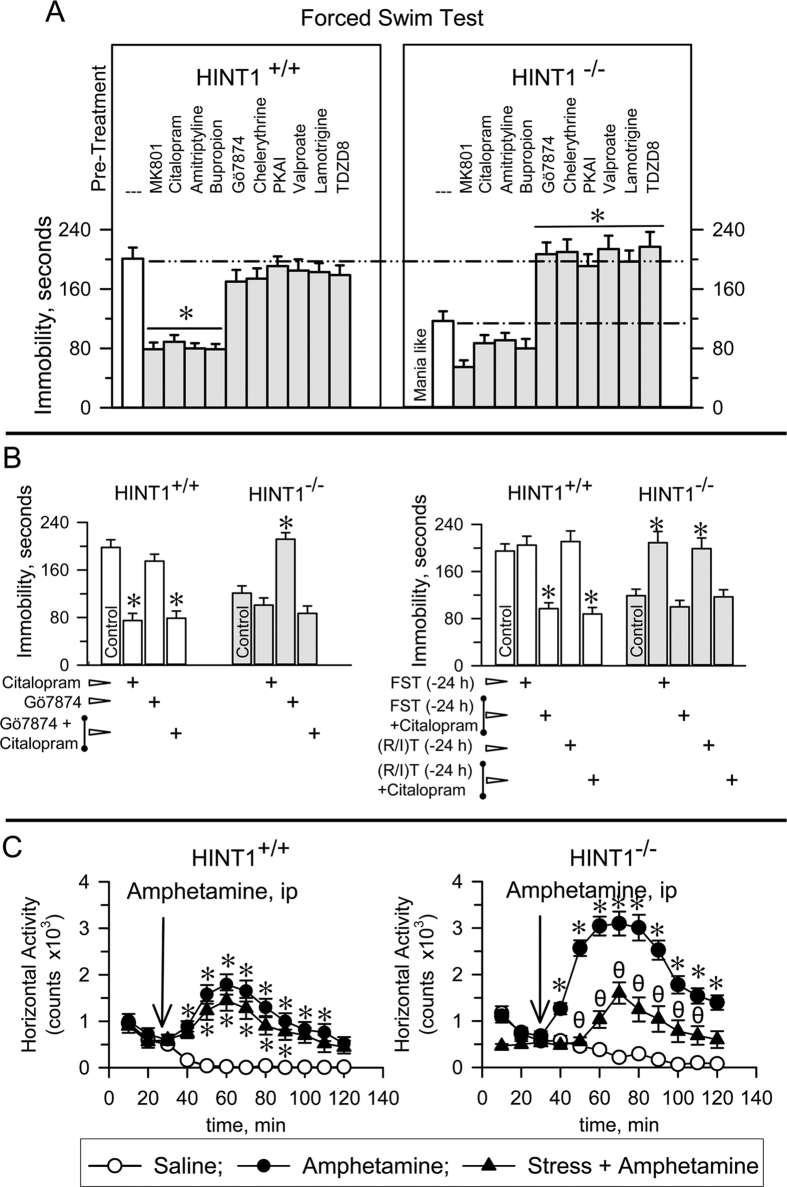
Swim activity of HINT1^+/+^ WT and HINT1^−/−^ mice during the FST: effects of stress, PKC and GSK3β inhibitors, antidepressants, and mood-stabilizers. (**A**) Compounds were acutely administered either systemically ip (citalopram, 10 mg/kg; amitriptyline, 5 mg/kg; bupropion, 10 mg/kg; valproate, 200 mg/kg; lamotrigine, 30 mg/kg; 60 min prior the FST) or icv (MK801, 1 nmol; Gö7874, 1 nmol; chelerythrine, 2 nmol; PKAi, 5 nmol; TDZD8, 20 nmol; 30 min prior the test). These doses did not significantly affect spontaneous mouse activity. (**B**) Effects of citalopram, PKC inhibition, FST exposure or resident intruder test (R/I) exposure on the immobility periods of HINT1^−/−^ (pro-depressive behavior) and HINT1^+/+^ WT mice during the FST. (**A**,**B)** The results are expressed as the mean ± SEM of the total scores (n = 8/group). *Indicate significant differences from the group treated with saline. ANOVA, Dunnett multiple comparisons vs control group, *p* < 0.05. (**C**) Effect of pre-exposure to acute stress on the motor-activating effects of the psychostimulant amphetamine in HINT1^+/+^ WT and HINT1^−/−^ mice. Each point is the computed mean ± SEM. *Significantly different from the control (saline) group (n = 8 per group); θ significantly different from the naïve group that received amphetamine. See Fig. 2A.

Consistent with their manic-like profile, HINT1^−/−^ mice exhibited greater swim activity and, consequently, shorter immobility periods during the FST than did HINT1^+/+^ WT mice. We then investigated in these mice the effects of pharmacological interventions that are effective in treating human mania and depression. Citalopram, amitriptyline, and bupropion did not produce behavior changes in HINT1^−/−^ mice but did increase the activity levels of HINT1^+/+^ WT mice such that their periods of inactivity were similar to those exhibited by HINT1^−/−^ mice. The NMDAR antagonist MK801 increased the struggling and swim activity levels of HINT1^+/+^ WT mice while slightly augmenting the more animated behaviors of HINT1^−/−^ mice during the FST (Fig. 3A). iPKCs, such as Gö7874 and chelerythrine, did not significantly alter the activity levels of HINT1^+/+^ WT mice at the doses used in this study. However, these drugs reduced the swim activity exhibited by HINT1^−/−^ mice to the levels observed for HINT1^+/+^ WT mice. PKA inhibitors, lamotrigine, valproate and GSK3β inhibitors did not affect the swim behaviors of HINT1^+/+^ WT mice; however, these drugs also mitigated the manic-like behaviors of HINT1^−/−^ mice. Interestingly, Gö7874 did not alter the activating effects of citalopram in HINT1^+/+^ WT mice; however, citalopram inhibited the effects of PKC inhibition in HINT1^−/−^ mice and thus preserved their manic-like behavior (Fig. 3B, left panel).

During the FST, development of depressive-like behaviors reduced the swim activity of HINT1^+/+^ WT mice, but this effect was less evident in HINT1^−/−^ mice. A second exposure of the mice to the stressful FST 24 h later did not significantly alter the activity of HINT1^+/+^ WT mice, but reduced the swim activity of HINT1^−/−^ mice (Fig. 3B, right panel). HINT1^−/−^ mice exhibited significantly better learning than wild type HINT1^+/+^ mice during passive avoidance testing and also exhibited signs of elevated neurogenesis compared with their counterparts (Supp. Fig. 2A,B). We thus considered the possibility that the abovementioned changes in their swim behavior may have been the result of learned behavior. However, it is worth noting that the activity levels of HINT1^−/−^ mice also decreased in the FST when these mice had previously been exposed to another form of stress, such as the resident-intruder test. Furthermore, when evaluated in the activity cage, HINT1^−/−^ and BStac mice exhibited higher activity than HINT1^+/+^ WT mice. As observed for the FST, the activity of HINT1^−/−^ mice in the activity cage subsequently diminished on day two and was comparable with that of HINT1^+/+^ WT mice, probably as a result of previous testing, while the activity levels of BStac mice remained elevated (Supp. Fig. 2C). Notably, citalopram restored manic-like symptoms in stress-primed HINT1^−/−^ mice (Fig. 3B right panel). Acute stress attenuated the activating effects of amphetamine in HINT1^−/−^ mice, but not in HINT1^+/+^ WT mice (Fig. 3C). In contrast to HINT1^−/−^ mice, BStac mice exhibited persistent manic-like behaviors after exposure to multiple forms of stress.

### Stress promotes depressive-like behaviors in HINT1^−/−^ mice

**Figure 4:**
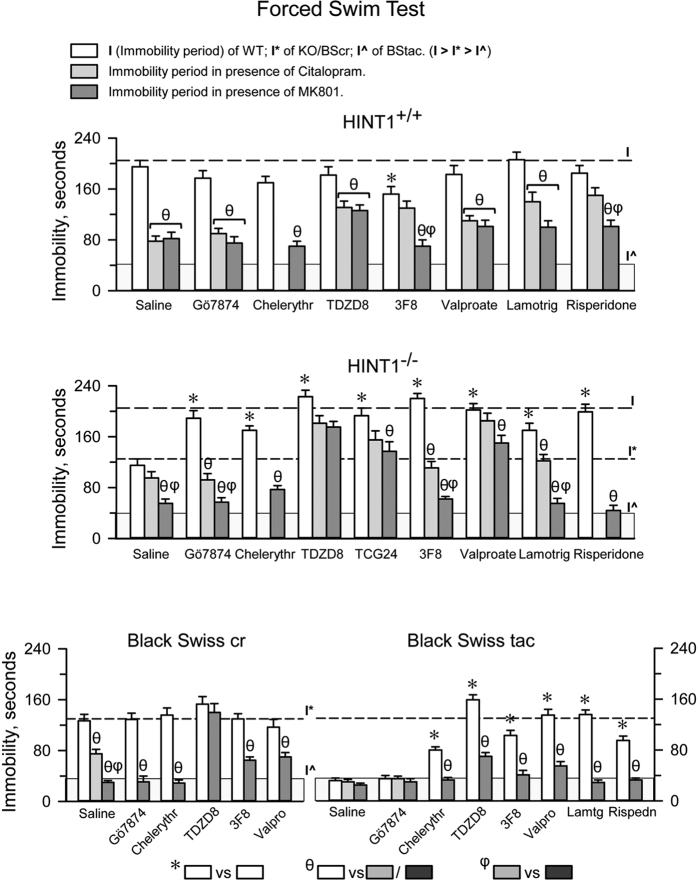
NMDAR antagonism abolishes depressive-like behaviors and unmasks anti-manic effects promoted by pharmacological treatments in mice during the FST. The effects of PKC inhibition, GSK3β inhibitors, and different mood stabilizers were studied in HINT1^+/+^ WT, HINT1^−/−^, BScr, and BStac mice treated with vehicle, MK801 (1 nmol, icv), or citalopram (10 mg/kg, ip). The compounds were administered as indicated in Fig. 3. Risperidone (7 nmol, icv) and 3F8 (1 nmol, icv) were injected 30 min prior to the FST. The doses of MK801 and citalopram used in this study produced comparable effects on the swim activity of HINT1^+/+^ WT mice. Each bar represents the mean ± SEM of the data obtained from 10 mice. The balance between activity-stimulating behaviors and pro-depressive behaviors determined the swim vigor exhibited by the animals during the FST. Lower immobility scores (I) implied higher mice vigor (swim activity). For each strain of mice, *indicates a significant difference from the control group, which received saline instead of treatment (open bars), θ indicates that the activity of the mice significantly increased after receiving MK801/citalopram (gray bars), and φ indicates that the effects of MK801 on the corresponding treatment are different from those of citalopram. ANOVA, Dunnett multiple comparisons vs control group, *p* < 0.05.

HINT1^−/−^ mice exhibited manic-like behaviors and greater swim activity in the FST than HINT1^+/+^ WT mice. Although the activity level of BScr mice was higher than that of HINT1^+/+^ WT mice, both groups may be considered control animals for BStac and HINT1^−/−^ mice respectively. First, BScr or HINT1^+/+^ WT mice did not exhibit significant behavior changes following exposure to acute stress or treatment with GSK3β inhibitors, mood stabilizers, lamotrigine, or risperidone. Second, the aforementioned pharmacological treatments increased swim immobility in HINT1^−/−^ mice to the levels of HINT1^+/+^ WT mice, and in BStac mice to the levels seen in BScr mice (Fig. 4).

In HINT1^+/+^ WT mice that had received saline or iPKCs, administration of MK801 or citalopram exerted similar stimulatory effects on their swim behavior. However, MK801/citalopram did not produce such stimulation when HINT1^+/+^ WT mice received TDZD8, valproate, or lamotrigine. MK801 was effective counteracting the effects of iPKCs, lamotrigine, or 3F8 on the swim activity of HINT1^−/−^ mice. However, MK801 was much less effective inhibiting the effects of valproate or other GSK3β inhibitors, such as TDZD8 and TGC24, in these mice. Thus, the expression of stress-evoked depressive-like episodes requires NMDAR activity, and NMDAR inhibition, citalopram and certain GSK3β inhibitors diminish the manifestation of these behaviors.

**Figure 5:**
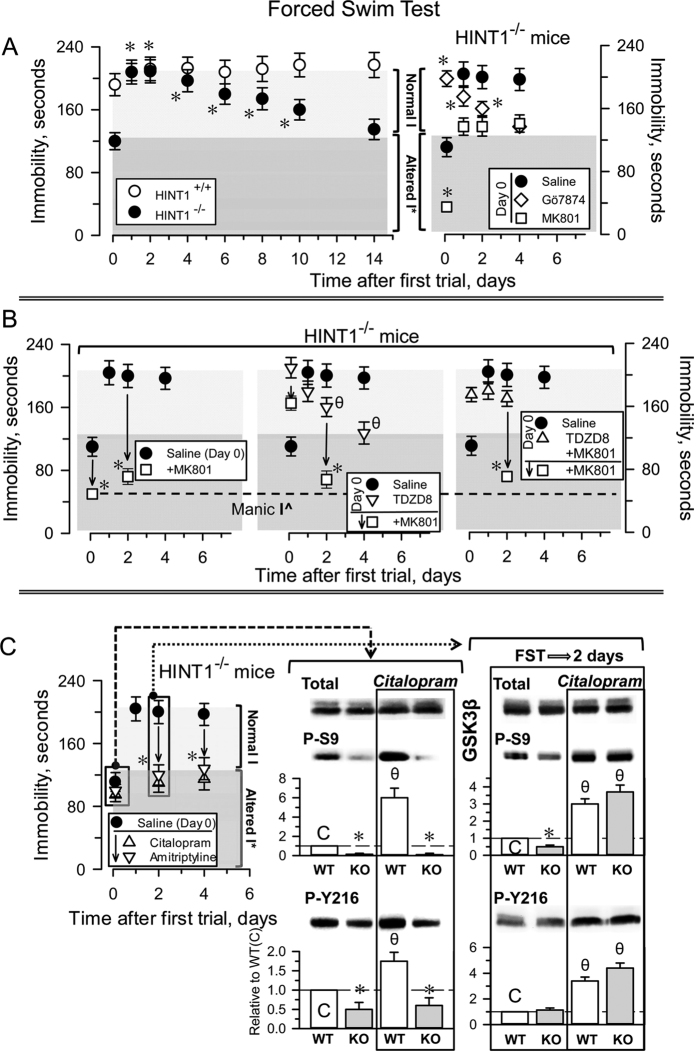
Behavioral and molecular changes induced by stress in HINT1^+/+^ WT and HINT1^−/−^ mice. (**A**) Left panel: Untreated mice were subjected to the FST at day 0 and were divided into groups of six. At each indicated time interval, a different group was re-evaluated. The immobility periods of HINT1^+/+^ WT (Normal I) and HINT1^−/−^ mice (Altered I*) during the first FST are indicated. Right panel: effects of MK801 and Gö7874 on the behavior exhibited by stress-primed HINT1^−/−^ mice. The results are the mean ± SEM of total scores (n = 6/group). *Significant difference from the immobility (I*) displayed by HINT1^−/−^ control mice on the initial FST (day 0). (**B**) Effects of MK801 and TDZD8 on the depressive-like behaviors exhibited by stressed HINT1^−/−^ mice. On day 0, the mice received the treatment indicated before the first FST. In the middle panel, θ indicates that after two days, swim activity of HINT1^−/−^ mice receiving saline was significantly lower than that of those receiving TDZD8. *MK801 given at the days specified by the arrows significantly increased mice swim activity during the second FST. The data are the mean ± SEM of total scores (n = 6/group). (**C**) Left panel: citalopram/amitriptyline administration reduced the immobility of stressed but not naïve HINT1^−/−^ mice (n = 6). *Significant difference from saline-treated HINT1^−/−^ mice at the indicated time after the first FST. Middle and right panels: effect of citalopram on GSK3β content and regulatory phosphorylation, P-S9 and p-Y216, in frontal cortices of HINT1^+/+^ WT and HINT1^−/−^ KO mice. Mice receiving saline or citalopram were immediately euthanized after the first FST (day 0) or after the second FST, which was performed 48 h after the first. Each bar represents the mean ± SEM of data from at least three determinations using different gels and blots. An arbitrary value of 1 was assigned to the control HINT1^+/+^ WT mice data (**C**). *Significant difference between the KO (gray bars) and the WT group (open bars); θ indicates significant differences caused by citalopram but within each group of mice, WT and KO. All comparisons: ANOVA, Dunnett multiple comparisons vs control group, *p* < 0.05.

Regarding HINT1^−/−^ and BStac mice, with the exception of iPKCs, interventions that reduced the swim activity of HINT1^−/−^ mice, such as TDZD8 and valproate, significantly increased the immobility of BStac mice during the FST. While MK801 and citalopram failed to enhance the swim activity of BStac mice, MK801 reduced the effects of TDZD8 and valproate in these “manic” mice. It seems that NMDAR-dependent processes cannot prevail over processes triggering manic-like behavior in BStac mice. In these mice, inhibition of GSK3β, but not acute stress, attenuated the expression of pro-manic mechanisms to the level that permitted the expression of depressive-like behaviors (Fig. 4). The behavior of acutely stressed HINT1^−/−^ mice was similar to that of HINT1^+/+^ WT mice for several days. However, over time, stressed HINT1^−/−^ mice gradually began to exhibit their previous manic-like behaviors, such as increases in their swim activity (Fig. 5A). In contrast, the effects of PKC or GSK3β inhibition on the swim activity of stressed HINT1^−/−^ mice persisted for only 2 or 3 days (Fig. 5A,B). Remarkably, antidepressants did not modify the swim activity of naïve HINT1^−/−^ mice; however, these drugs rescued the manic-like swim activity of stressed HINT1^−/−^ mice exhibiting depressive-like behaviors (Fig. 5C). The NMDAR antagonist MK801 also increased the activity levels of stressed HINT1^−/−^ mice but to the extent that activity levels were similar to those exhibited by “manic” BStac mice (Fig. 5B). Notably, a single administration of the NMDAR antagonist prior the first FST prevented stress from promoting depressive-like behaviors during the days that followed (Fig. 5A).

### HINT1^−/−^ mice exhibit increased PKC activity and altered glutamate NMDA receptors

**Figure 6:**
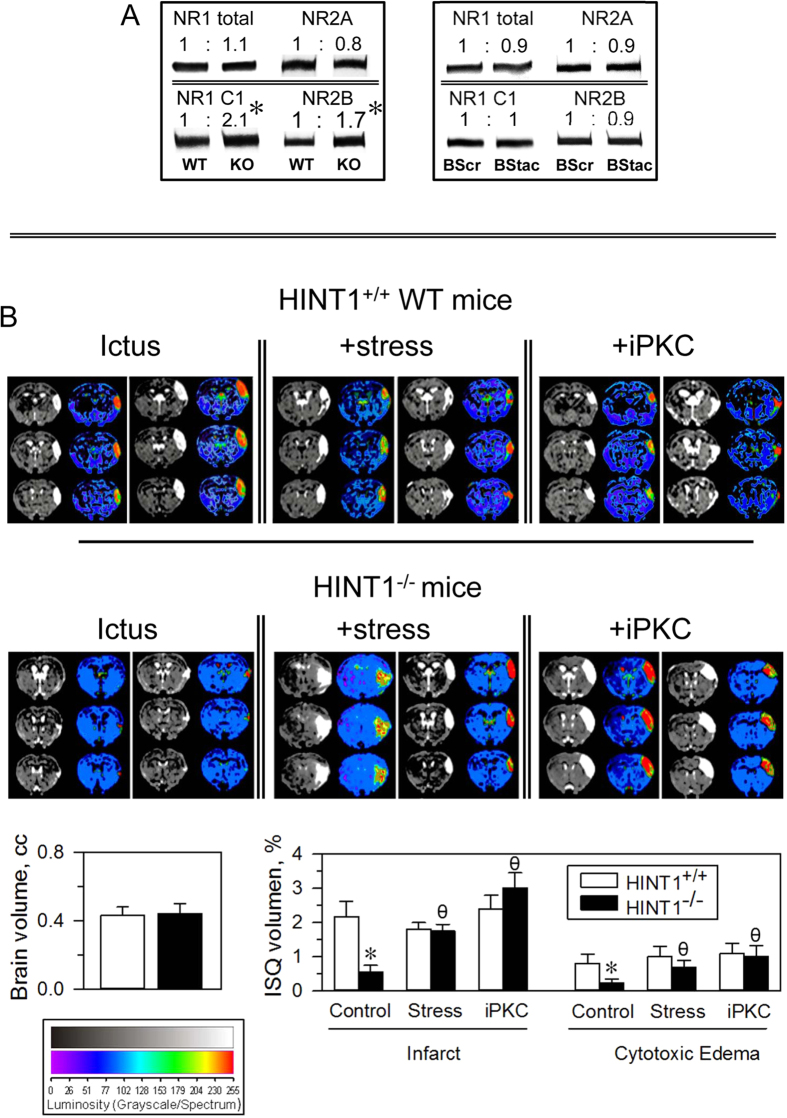
(**A**) NMDAR NR subunits in frontal cortices of HINT1^+/+^ (WT), HINT1^−/−^ (KO), BScr, and BStac mice. Within each row, the values of KO were referred to that of WT mice (assigned an arbitrary value of 1). Equal for BStac vs BScr mice. *Significantly different from the control group, ANOVA, Dunnett multiple comparisons vs control group, *p* < 0.05. Representative blots are shown (details, see Fig. 1). (**B**) Effect of PKC inhibition and stress on ischemic brain damage elicited in HINT1^−/−^ mice. Representative brain section images from HINT1^+/+^ WT and HINT1^−/−^ mice obtained 48 h after pMCAO (BIOSPEC BMT 47/40 (Bruker, Ettlingen, Germany). Mice were stressed (FST) or treated with Gö7874 (1nmol, iPKC) prior to surgery. The dorsal third ventricle was used as an internal anatomical marker in HINT1^+/+^ WT and HINT1^−/−^ mice to align, register, and compare the image collections from each mouse. The infarct volume was calculated as the percentage of the hemisphere that was infarcted (ImageJ 1.44 l software, NIH, Bethesda, MD, USA). The groups comprised eight mice, and the data are represented as the mean ± SEM. The bar graphs indicate the edema and infarct volumes (mean ± SEM) of the HINT1^+/+^ WT (white bars) and HINT1^−/−^ (black bars) mice. *Significantly different from the paired HINT1^+/+^ WT group; ϕ significantly different from the HINT1^−/−^ control group; paired *t* test, *p* < 0.05.

HINT1^−/−^ mice share a series of therapeutically relevant alterations with BPD patients, such as increased PKC, PKA and GSK3β activity levels and altered glutamate NMDAR composition and function. Although the levels of NMDAR NR1 subunit were similar in cortical synaptosomes of HINT1^−/−^ and HINT1^+/+^ WT mice, the levels of the NR1 subtype that carries the C1 cytosolic segment were two-fold higher in HINT1^−/−^ mice. NR2A subunit levels were comparable in these two groups of mice, but NR2B subunit levels increased in HINT1^−/−^ mice. Notably, BStac and BScr mice exhibited no differences in NMDAR subunit levels (Fig. 6A).

The possible influence of NR2B-enriched NMDARs on synaptic function was then addressed. The electrophysiological analysis indicated that synaptic properties were apparently similar in HINT1^−/−^ and HINT1^+/+^ WT neurons. Thus, stimulation-evoked excitatory postsynaptic currents (EPSCs) did not significantly differ between these neurons. However, whole-cell recording from CA1 pyramidal neurons detected NMDAR-mediated slow inward currents of higher amplitude in HINT1^−/−^ neurons. Moreover, the NMDAR/AMPAR ratio of the EPSCs increased in HINT1^−/−^ neurons, and interestingly, as a consequence of PKC inhibition, paired pulse facilitation increased in HINT1^−/−^ mice, but not in HINT1^+/+^ WT mice, suggesting an enhanced neurotransmitter release in HINT1^−/−^ mice (Supp. Fig. 3). Neural facilitation refers to a condition whereby postsynaptic potentials increase when a stimulatory impulse closely follows a previous and identical impulse. This paired pulse facilitation is a form of short-term synaptic plasticity mediated exclusively by pre-synaptic neurotransmitter release. Therefore, in HINT1^−/−^ mice an apparent synaptic normality covers a series of molecular and physiological abnormalities, such as the abovementioned increases in PKC activity, altered NMDAR vs AMPAR function and an increased NR2B/NR2A subunit ratio.

Oscillations in the functional state of cortical synapses are essential for processes such as learning, memory consolidation and storage. Relevant to our study, the adequate transition from long-term potentiation (LTP) to long-term depression (LTD), and vice versa, requires the coordinated activities of GSK3β and NMDARs. Alterations in their function elicit a series of synaptopathies that are associated with different neural disorders, including BPD. The ratio of P-Y216 to P-S9 was higher in HINT1^−/−^ mice than in HINT1^+/+^ WT mice (Figs 2B and 5C). Citalopram enhanced P-S9 and P-Y216 GSK3β levels in HINT1^+/+^ WT mice but surprisingly failed to do so in HINT1^−/−^ mice. Exposure of the latter mice to emotional stress resulted in increased GSK3β phosphorylation. In these circumstances, citalopram altered the levels of GSK3β regulatory phosphorylations in HINT1^−/−^ mice (Fig. 5C). The NMDAR blocker MK801 was more effective than citalopram in increasing swim activity and GSK3β phosphorylation levels in HINT1^−/−^ mice (Supp. Fig. 4).

PKC activity increases in BPD patients and thus, the study of this kinase in this illness has gained relevance in recent years^35^,^36^. HINT1^−/−^ mice exhibit high PKC activity levels but also increased NMDAR responses to their direct activators^19^,^20^. Thus, suggesting an adaptive response of NMDARs to prevent glutamate hypofunction in these mice. Inhibition of PKC in these mice increased their mobility, via MK801-sensitive behaviors. Because the HINT1 protein negatively regulates PKC and NMDAR activity^20^,^37^, the possibility exists that PKC restrains NMDAR activity in HINT1^−/−^ mice. We addressed this issue using the excitotoxic neurological damage produced by stroke, which is mostly mediated by NMDAR overactivation^38^. HINT1^−/−^ mice subjected to unilateral permanent middle cerebral artery occlusion (pMCAO) exhibit much less severe infarction and edema than are seen in HINT1^+/+^ WT mice^22^. We could observe that PKC inhibition prior to surgery did not affect stroke-induced neurological damage severity in HINT1^+/+^ WT mice; however, this intervention greatly enhanced stroke-induced brain damage in HINT1^−/−^ mice (Fig. 6B). Because acute stress and PKC inhibition exerted comparable effects on the swim behavior of HINT1^−/−^ mice, we also evaluated the influence of stress in this animal model of glutamate-mediated ischemic damage. Previous exposure of mice to the FST brought about increases in stroke-induced neural damage in HINT1^−/−^ mice but did not augment its severity in HINT1^+/+^ WT mice (Fig. 6B). Similar to the electrophysiological study, the stroke assay also suggested that PKC negatively regulates NMDARs in HINT1^−/−^ mice and that PKC inhibition or acute stress removes this control.

### PKC regulates the Akt/GSK3β signaling pathway in HINT1^−/−^ mice

**Figure 7:**
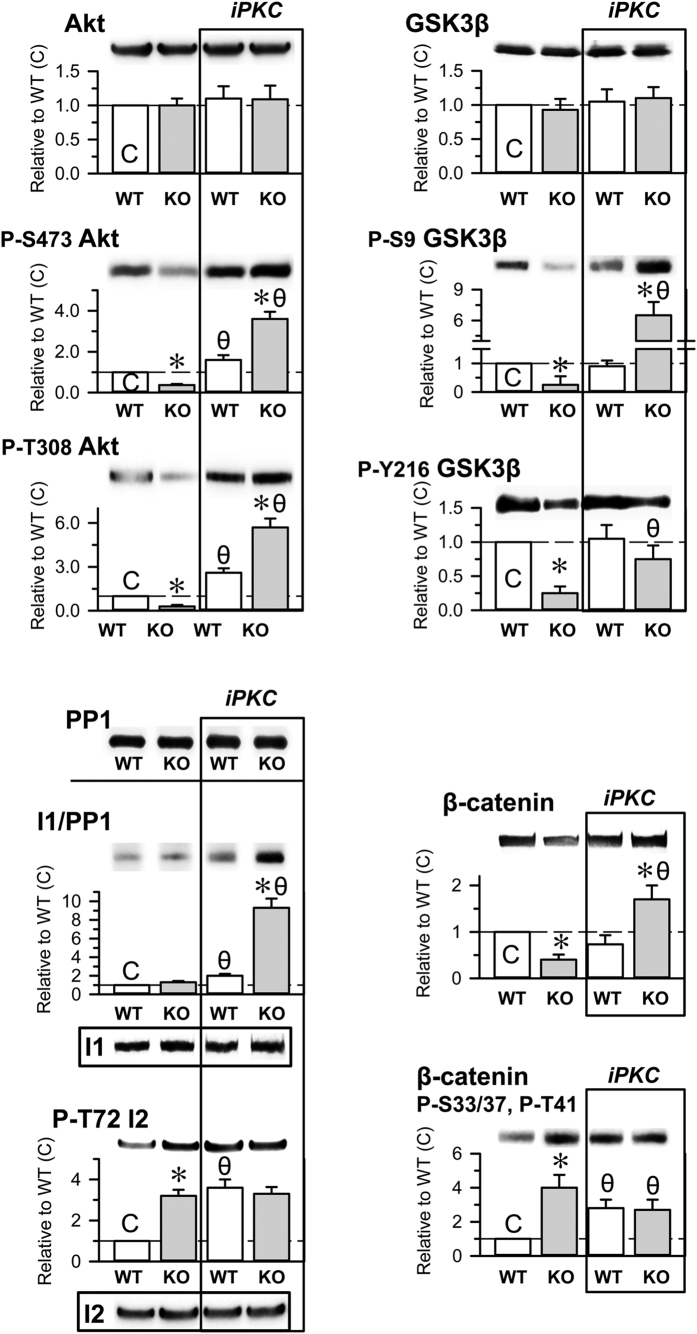
Effects of PKC inhibition on the Akt/GSK3β signaling pathway in HINT1^+/+^ WT and HINT1^−/−^ KO mice. The iPKC Gö7874 (1 nmol, icv) was injected 30 min before euthanasia. The levels of the signaling proteins and of their phosphorylated forms were determined in synaptosomes obtained from mouse prefrontal cortices. Representative blots are shown. Tubulin was used as a loading control. Each bar represents the mean ± SEM of the data from at least three determinations, which were performed using different gels and blots. An arbitrary value of 1 was assigned to the control HINT1^+/+^ WT data (**C**). *Significant difference between the KO (grey bars) and the WT group (open bars); θ indicates a significant differences caused by Gö7874 but within each group of mice, WT and KO. ANOVA, all pairwise Holm-Sidak multiple comparison tests, *p* < 0.05.

**Figure 8:**
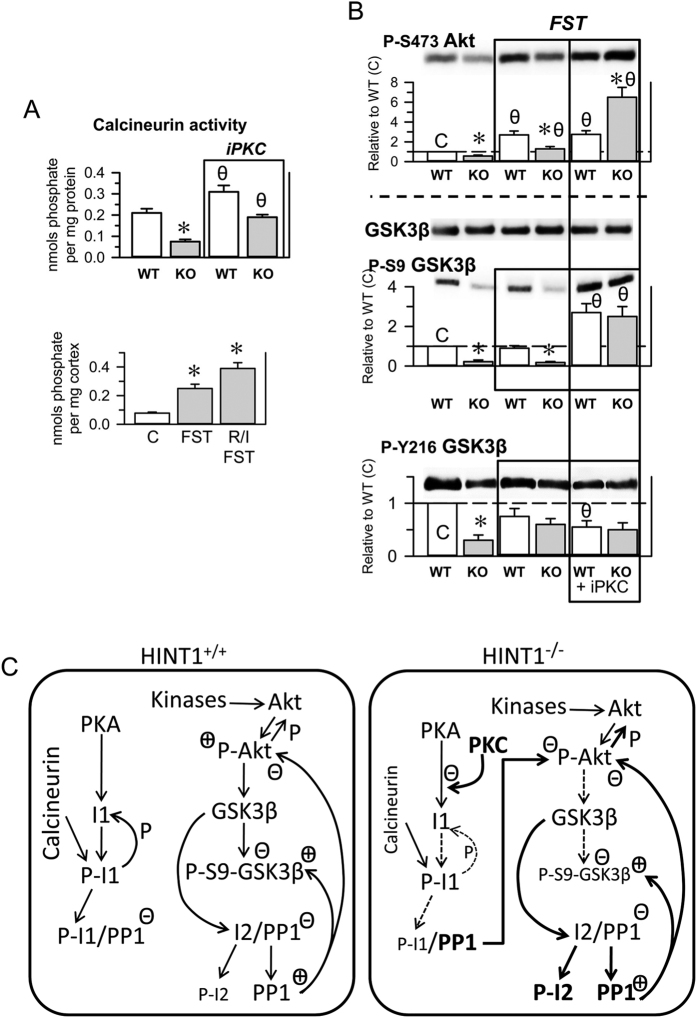
The effects of acute stress and PKC inhibition on calcineurin and Akt/GSK3β signaling pathway. (**A**) Calcineurin enzymatic activity in prefrontal brain lysates from HINT1^+/+^ WT and HINT1^−/−^ KO mice (n = 6). The effects of iPKC (upper panel) or stress (FST, R/I) (lower panel) on enzymatic activity were evaluated. *Significant difference from HINT1^+/+^ WT/C. θ Significant difference with respect to the corresponding group not receiving iPKC. ANOVA, Dunnett multiple comparisons vs control group, *p* < 0.05. (**B**) Mice that received vehicle or iPKC Gö7874 (1 nmol, icv) were immediately euthanized after the FST, and their frontal cortices were processed for molecular analysis. *For each situation, control, FST and FST + iPKC, the data of KO mice were compared with those of WT mice. θ The data from WT and KO mice (FST and FST + Gö7874) were compared with those of naïve control mice. For the statistical analysis and additional details, see Fig. 7 and the Methods section. (**C**) Diagram showing altered Akt/GSK3 signaling pathways in HINT1^−/−^ mice. Key: PP1 (serine and threonine protein phosphatase 1), I1 (PP1 inhibitor 1), I2 (PP1 inhibitor 2), P- (phosphorylated forms), P-I1/PP1 (I1-dependent PP1 inhibition), I2/PP1 (I2-dependent PP1 inhibition), + and − (facilitation and inhibition of enzymatic activities). For details, see the main text.

Alterations in the Akt/GSK3β signaling pathway have been associated with mood disorders and schizophrenia. Both proteins display kinase activity, and Akt reduces GSK3β activity by phosphorylating S9. HINT1^−/−^ mice show little phosphorylation at this GSK3β residue (Fig. 2B), and decreases in the levels of P-S9 GSK3β are correlated with the predominance of mania/hypomania in BPD patients^34^. Total Akt levels were comparable between HINT1^+/+^ WT mice and HINT1^−/−^ mice; however, the phosphorylations that activate Akt, S473 and T308, were present at much lower levels in HINT1^−/−^ mice. In this signaling pathway, protein phosphatase 1 (PP1) removes the abovementioned Akt and GSK3β phosphorylations. The activity of PP1 is negatively regulated by binding to the phosphorylated form of the inhibitor I1 and to the non-phosphorylated form of the inhibitor I2. Thus, Akt and GSK3β phosphorylation levels suggest that PP1 activity increased in HINT1^−/−^ mice. Whereas PP1, I1 and I2 levels were comparable in WT and KO mice, in KO mice the phosphorylation of inhibitor I2 was augmented, which may indeed promote PP1 activity (Fig. 7). The I2 is a substrate of GSK3β, and its increased phosphorylation correlates with the apparent higher activity of this kinase in HINT1^−/−^ mice. PKC and the calcium and calmodulin-regulated phosphatase calcineurin can also promote PP1 activity. PKC diminishes PKA-mediated phosphorylation of I1, and calcineurin removes this phosphorylation from I1. In HINT1^−/−^ mice P-I1/PP1 inhibitory complexes did not significantly differ from those observed in HINT1^+/+^ WT mice. PKC activates Cabin/Cain^39^, an inhibitor of calmodulin and in HINT1^−/−^ mice the activity of calcineurin diminished (Fig. 8A). Thus, the outcome of an increased PKC activity could compensate calcineurin negative effects on the levels of P-I1. β-catenin, another relevant signaling protein downstream of the Akt/GSK3β pathway, also influences the pathophysiology of neural disorders. In HINT1^−/−^ mice, the GSK3β-mediated phosphorylation of β-catenin that promotes its destruction increased, and β-catenin total levels were subsequently diminished (Fig. 7).

PKC negatively regulates the phosphatidylinositol 3-kinase/Akt signaling pathway^40^. Hence, we analyzed whether molecular changes exhibited by HINT1^−/−^ mice were due to increased PKC function. The inhibition of PKC did not alter the content of Akt, GSK3β, PP1, I1 or I2 in HINT1^+/+^ WT and HINT1^−/−^ mice. However, iPKC increased total levels of β-catenin in HINT1-deficient mice. PKC inhibition caused significant changes in the phosphorylation levels of the signaling proteins studied; P-S473 and P-T308 Akt were moderately augmented in HINT1^+/+^ WT mice and greatly increased in HINT1^−/−^ mice. In HINT1^+/+^ WT mice, iPKC did not alter GSK3β regulatory phosphorylation levels. In contrast, in HINT1^−/−^ mice, the activating P-Y216 GSK3β was augmented 3-fold and the inhibitory P-S9 GSK3β (Akt site) increased by 22-fold. A similar outcome was obtained for the inhibitory complex P-I1/PP1, which increased approximately 10-fold in HINT1^−/−^ mice, confirming the relevance of PKC as a brake for P-I1 formation in this signaling pathway. In accordance with the effect of iPKC on GSK3β phosphorylation, in HINT1^−/−^ mice, β-catenin exhibited reductions in P-S33/37 and P-T41 (GSK3β sites) and β-catenin total levels augmented. These results suggest an essential role for PKC in the molecular alterations observed in HINT1-defficient mice.

HINT1^−/−^ mice and HINT1^+/+^ WT mice responded differently to emotional stress paradigms, and these differences affected various biochemical parameters (Fig. 8). PKC activity negatively regulates calcineurin phosphatase activity; indeed, iPKC enhanced its effects in HINT1 KO and WT mice. Moreover, a few min after FST or resident/intruder tests, calcineurin activity increased in HINT1^−/−^ mice (Fig. 8A). After 6 min of the FST, P-S473 Akt levels increased in HINT1^+/+^ WT mice, but only slightly in HINT1^−/−^ mice. Equally, after this brief interval post-FST, P-S9 and Y216 GSK3β levels did not significantly change in either group. However, after 24/48 h, HINT1^−/−^ mice exhibited significant increases in GSK3β phosphorylation at both positions, approaching the levels observed in HINT1^+/+^ WT mice (Fig. 5C). Thus, in HINT1^−/−^ mice, the inhibition of PKC prior to the FST challenge precipitated changes in Akt/GSK3β phosphorylation levels similar to those observed when FST trials were spaced 24/48 h apart (Fig. 8B).

## Discussion

A suitable animal model of human BPD must exhibit molecular and behavioral alterations similar to those reported in BPD patients, and, most importantly, pharmacological interventions should stabilize the behavior of affected individuals without altering that of healthy individuals. The present study addressed these essential issues, and HINT1^−/−^ mice exhibited behavioral and molecular alterations paralleling those described in BPD patients. Human symptomatic treatments, such as mood stabilizers and PKC or GSK3β inhibitors, mostly affected the behavior of HINT1^−/−^ mice but had little or no effect on the behavior of HINT1^+/+^ WT mice.

Drug- and stress-free HINT1^−/−^ mice exhibited manic-like behaviors, such as those described in BStac mice, an animal model of human mania^41^. At the molecular level, manic-like behaviors manifest in conjunction with increases in GSK3β activity^34^. In our study, GSK3β inhibitors, mood stabilizers, and agents such as lamotrigine or risperidone did not alter the behavior of HINT1^+/+^ WT mice but reduced the struggling and increased the immobility of HINT1^−/−^ and BStac mice during the FST. A growing body of evidence suggests that GSK3β activity can be a suitable marker of mood diseases. At present, there is no direct and reliable method for evaluating GSK3β activity; thus, its regulatory phosphorylations provide us with an index of its enzymatic activity.

Negative regulation of GSK3β activity hinges primarily on Akt-mediated phosphorylation of GSK3β at S9, whereas enhancement of its basal activity hinges on its phosphorylation at Y216^33^. One should be cautious when estimating GSK3β activity based on solely its S9 inhibitory phosphorylation because increases in substrate can override Akt-mediated GSK3β inhibition^42^ and can thus lead to erroneous conclusions. In fact, the behavioral effects promoted by drugs or stressors that increase P-S9 GSK3β levels usually diverge from those of direct inhibitors of GSK3β function. Our study has provided several examples of such divergences. Thus, P-S9 GSK3β was virtually nonexistent in HINT1^−/−^ mice but this phosphorylation was elevated in BStac mice, and both classes of mice showed manic-like behaviors typical of increased GSK3β function. Citalopram and MK801 enhanced inhibitory P-S9 GSK3β levels but increased the swim activity HINT1^+/+^ WT mice in the FST. In contrast, direct inhibitors of GSK3β, such as TDZD8 and TCG24, did not exert such activating effects in these HINT1^+/+^ WT mice. PKC inhibition and acute stress increased P-S9 GSK3β levels, and this time promoted depression-like behaviors diminishing HINT1^−/−^ mice’s swim activity. However, in HINT1^−/−^ mice previously exposed to stress, citalopram and MK801, but not GSK3β inhibitors, further increased inhibitory P-S9 GSK3β levels but inhibited NMDAR-mediated pro-depressive conducts, thereby unveiling manic-like behaviors in these mice.

The aforementioned behavioral observations correlate better with the estimation of GSK3β activity whether we take into account the simultaneous changes promoted by those treatments in P-S9 and P-Y216 GSK3β levels. Y216 phosphorylation increases GSK3β activity by approximately 200-fold^33^; thus, this phosphorylation can increase GSK3β enzymatic function, even in the setting of increases in P-S9 levels, such as those promoted by citalopram and MK801. Taking into account both phosphorylated forms of abovementioned behavioral changes, the activity of this enzyme in manic-like mice would be higher than in “control mice”, *e.g*., “manic” BStac mice vs mice, and “manic” HINT1^−/−^ mice vs HINT1^+/+^ WT mice. Apart from the phosphorylation analysis, β-catenin levels BScr may serve as a marker of GSK3β activity. GSK3β phosphorylates β-catenin and promotes its degradation, which correlates with reductions in neurogenesis and the onset of depressive episodes. Unfortunately, a series of physiological alterations, such as those that occur in HINT1^−/−^ mice, compromise the suitability of β-catenin as a marker of GSK3β activity. HIT family proteins, such as HINT1 and FHIT, inhibit β-catenin transcriptional activity^43^; thus, in HINT1^−/−^ mice, increases in β-catenin activity may balance the negative effects of increased GSK3β activity. In fact, we observed enhanced neurogenesis in HINT1^−/−^ mice exhibiting low P-S9 GSK3β levels and decreased β-catenin levels. Increases in β-catenin transcriptional activity and PKC activity in these mice probably contributed to this finding^44^.

The FST has been particularly useful for characterizing the differential behaviors and responses to treatment exhibited by HINT1^−/−^ mice. In this stressful paradigm, mice must overcome an aversive situation but must also avoid excessive exertion, which may be harmful. The balance between swim activity and its negative regulation (obstructing mechanisms) drives mouse behavior during this test. In humans, depression is associated with increased glutamate NMDAR activity, whose antagonism by ketamine leads to rapid, robust, and relatively sustained antidepressant effects in patients with treatment-resistant major depression that contrast with the delayed effects observed with the use of traditional antidepressants^17^,^45^. Accordingly, drugs with antidepressant activity in humans and NMDAR blockers do not promote activity. On the contrary, they impede development of the mechanisms counteracting activity, resulting in enhanced mouse swim activity during the FST.

Comparative study of the behaviors exhibited by HINT1^−/−^ and BStac mice suggested the existence of a cross-regulatory relationship between the molecular mechanisms underpinning manic-like behaviors and those supporting depressive-like behaviors. A series of observations led us to propose this connection. For example, during the FST, HINT1^+/+^ WT mice and BScr mice exhibited citalopram- and MK801-sensitive depressive-like behaviors. However, “manic” BStac mice did not exhibit these behaviors. HINT1^−/−^ mice showed depressive-like behaviors, albeit to a limited extent. HINT1^−/−^ mice previously exposed to paradigms such as the FST, the TST, the resident-intruder test and even the activity cage exhibited a higher capacity to express such depressive-like behaviors than did the other mice. Therefore, BStac and HINT1^−/−^ mice exhibited manic-like behaviors in response to stress; however, only the latter mice developed depression-like behaviors. Long-term suppression of pro-depressive behaviors may facilitate the appearance of manic symptoms, similar to the case in depressive patients who may display such signs following long-term administration of antidepressants^46^,^47^. Indeed, stressful environmental factors are believed to trigger the depressive phase in BPD patients, and tricyclic antidepressant use during depressive episodes can precipitate the expression of mania^48^,^49^. In support of using HINT1^−/−^ mice as a model for human BPD, MK801 and, to a lesser extent, citalopram by reducing depression-like behaviors increased the activity of HINT1^+/+^ WT mice and rescued the manic-like state in stressed HINT1^−/−^ mice. It is possible that by negating the depressive-like component of mouse behavior, these drugs increase the activity of HINT1^+/+^ WT mice and unveil the manic-like hyperactivity of HINT1^−/−^ mice. Following this reasoning, the manic-activity of BStac mice prevents stress from triggering the mechanisms necessary to promote depressive-like behaviors. Alternatively, stress may trigger these mechanisms, but depressive-like behaviors barely express because of the quality of the manic-like behavior in these mice. Drugs reducing manic-like behaviors, such as mood stabilizers and GSK3β inhibitors, diminished the swim vigor of BStac mice, thereby increasing their immobility periods in the FST. Importantly, MK801 revealed that BStac mice develop depressive-like behaviors under these circumstances.

It is therefore feasible that neural processes stimulating and obstructing the activity of the individual do not actually alternate but instead compete for predominance. In a normal subject, the outcome of this antagonism molds its conduct to fit the changing environment; however, if the strength of one or both antagonists compromises the real-time adaptation of the subject to the milieu, symptoms of mania, depression of even bipolar disorder may arise. In “manic” BStac mice, the strength of the mechanisms that promote activity predominates over the strength of the antagonists; however, in HINT1^−/−^ mice, stress triggers delayed depressive-like behavior. Thus, after several hours of being exposed to stressful situations, HINT1^−/−^ mice gradually behaved in a manner similar to that of HINT1^+/+^ WT mice in different experiments, including the FST and TST. Thereafter, HINT1^−/−^ mice rapidly manifested depressive-like behaviors in response to subsequent stressful situations. This acquired capacity persisted for a couple of weeks thereafter. Thus, testing of HINT1^−/−^ mice may bias their behavior in successive behavioral tests^19^,^26^^, and present study^. To circumvent this drawback, with the exception of mice that we evaluated to determine the effects of acute stress on subsequent test outcomes, we used HINT1^−/−^ and HINT1^+/+^ WT control mice only once.

Our behavioral data support the use of HINT1^−/−^ mice as a model for studying and detecting drugs that have potential as treatments for human BPD. In addition, the fact that their molecular alterations parallel those described in BPD patients suggest that this model has potential as a means of studying certain functional aspects of this neural disease. In BPD, increases in PKC activity apparently contribute to the manic phase. Recent clinical assays involving the relatively selective iPKC tamoxifen have confirmed the importance of this target in BPD therapy^10^. PKA activity is also increased in bipolar mania patients and decreased in depressed patients^50^. Accordingly, in this study, HINT1^−/−^ mice exhibited augmented PKC and PKA activity. Inhibition of these kinases reduced manic-like behaviors, thereby increasing the immobility of HINT1^−/−^ mice during the FST and TST. BStac mice also exhibited an increased PKC activity; however, its inhibition did not significantly affect the behavior of these “manic” mice during the FST^29^^, and present study^. Thus, enhanced PKC activity does not necessarily support human mania but may support other features of the bipolar disorder^14^,^51^. This interesting possibility raises the question of which mechanisms powered by PKC enable the kinase to facilitate such complex activity.

In mania, GSK3β, PKC and PKA activity levels increase, but only GSK3β inhibitors reduced the swim activity displayed by BStac and HINT1^−/−^ mice in the FST. PKC inhibition and emotional stress caused depressive-like behaviors only in HINT1^−/−^ mice, suggesting that additional physiological alterations account for this critical difference. Neuropathological studies revealed decreased expression of NR2A subunit transcripts in BPD patients^12^. Although the levels of NR2A subunits were comparable between HINT1^+/+^ WT and HINT1^−/−^ mice, the levels of NR2B subunits were significantly increased in HINT1^−/−^ mice. The calcium channels of NMDARs containing NR2B, NR2C, or NR2D subunits have a lower probability of being open than those of NR2A-containing NMDARs. Thus, NR2B-containing NMDARs exhibit slower kinetics^52^ and make larger contributions to synaptic LTD, whereas NR2A/2B-containing NMDARs mostly mediate synaptic LTP^53^,^54^. GSK3β also plays an essential role in establishing this synaptic plasticity. Inhibition of GSK3β favors NMDAR-mediated LTP, and activation of GSK3β favors the LTD state^55^. Thus, in HINT1^−/−^ mice, synaptic LTD prevails because of increases in the levels of NR2B subunits, inhibitory effects exerted by NR2B-containing NMDARs on NR2A-containing NMDARs^56^, and increases in GSK3β activity. The predominance of NR2A-containing NMDARs causes depressive-like episodes, while that of NR2B-containing NMDARs mainly causes manic-like episodes. Targeted deletion of the *NR2A* gene provokes manic-like behaviors in mice^57^. These behaviors are spontaneously present in HINT1^−/−^ mice^26^^, and present study^. In contrast, the levels of NR2A subunits are increased in the lateral amygdala of depressed patients^58^ and in isolated rats^59^ a rodent model for antidepressant detection^60^.

BPD patients exhibit more creativity than healthy individuals^61^, and both BPD I and BPD II patients commonly exhibit this trait during hypomanic/manic episodes^62^. BPD patients exhibit poor performance with respect to visuospatial reasoning; however, they often exhibit high intellectual performance^63^,^64^. Although these parameters are difficult to extrapolate to rodents, we have observed that NR2B-enriched HINT1^−/−^ mice exhibit increased passive-avoidance learning. BPD patients are essentially manic, but in response to environmental triggers, they can develop depression. This process requires concurrent malfunctions related to PKC and NMDAR activity. PKC regulates the function of NMDARs by direct serine phosphorylation of NR1 and NR2 subunits and via PKC-regulated Src-mediated tyrosine phosphorylation of NR2 subunits^65^. HINT1 binds to PKC and controls its activity; thus, *HINT1* deletions facilitate enhanced PKC activity and subsequent alterations in NMDAR activity, both of which relate to the pathophysiology of human BPD.

Therefore, alterations at NMDAR level apparently facilitate the differential responses exhibited by BStac and HINT1^−/−^ mice in response to stressful paradigms and pharmacological interventions. In the latter group of mice, the NR2B/NR2A and NMDAR/AMPAR ratios increase, resulting in a predominance of NMDARs containing NR2B subunits and impaired LTD/LTP^66^. PKC and NMDARs are important for neuronal development and circuit formation. Inhibition of PKC, or blockage of NMDARs, impairs the refinement of synaptic contacts suggesting that PKC participates in NMDAR-driven stabilization of developing synapses^67^. The expression of NMDAR NR2 subunits changes during development, and at birth NR2B expresses at nearly adult levels and in cerebral cortex has a dominant role in NMDAR function. In the following weeks, and in response to environmental stimuli NR2A levels increase throughout the brain^68^. NR2A and NR2B have both overlapping and distinct functions in dendritic development^69^, and the switch in synaptic NR2A subunit predominance implies the formation of new synapses from which NR2B is lacking^70^. PKC by increasing the probability of channel opening enhances the function of NR2A- but not of NR2B-containing NMDARs^71^. In this scenario, during brain development an abnormal high PKC function may reduce expression of its target, NR2A subunits, resulting in predominance of NR2B-enriched NMDARs. In late stages, these changes would produce alterations in brain circuitry and development such as these causing the BPD. In fact, reductions on NR2A subunit expression such as those observed in animal models of traumatic injury to the immature brain, impairs experience-dependent neuroplasticity^72^.

HINT1 proteins coordinate with sigma 1 receptors to support cross-regulation between GPCRs and glutamate NMDARs. Only NMDARs carrying NR1 subunits with the cytosolic C1 segment can couple with GPCRs via HINT1 proteins^22^. The lack of HINT1 proteins impairs the capacity of GPCRs to enhance NMDAR function. Thus, in HINT1^−/−^ mice, increases in NR1 C1 subunits and higher sensitivity to synaptic activators apparently compensate for NMDAR hypofunction^20^^, and present study^. In these mice, increased PKC activity augments GSK3β function, which, among other effects, diminishes the presynaptic release of glutamate, as well as that of other neurotransmitters^55^. Acute stress, PKC inhibition and certain GSK3β inhibitors increase neurotransmitter release, causing overactivation of their altered NMDARs and the subsequent development of depression-like behaviors. The pMCAO ictus model revealed the presence of NMDAR hypofunction in HINT1^−/−^ mice and demonstrated an increase in the sensitivity of these glutamate receptors to their specific activators^20^,^22^^, and present study^. MK801 prevented emotional stress from precipitating depressive-like behaviors in HINT1-deficient mice. Similarly, ketamine and memantine can counteract depressive symptoms and reduce mood cycling in BPD patients^73^,^74^,^75^, suggesting that these patients may suffer from alterations in NMDAR function comparable to those observed in HINT1^−/−^ mice. Inhibition of GSK3β by TDZD8 or TCG24 reduced the manic-like activity of HINT1^−/−^ mice. In these circumstances, citalopram or MK801 affected their modified behavior to only a limited extent. It is therefore possible that reductions in the activity of pro-manic mechanisms cause balance between the physiological processes promoting and obstructing mouse activity. Thus, inhibition of GSK3β by weakening stress-induced activity mechanisms indirectly reduces the reactive NMDAR-mediated depressive-like behaviors.

The calcium and calmodulin-dependent phosphatase calcineurin can diminish P-S9 GSK3β levels, and thus exert pro-mania effects. However, the activity of this phosphatase was reduced in HINT1^−/−^ mice, probably because of PKC-mediated activation of its inhibitor Cabin/Cain^39^. Thus, PKC can regulate the levels of inhibitory P-S9 GSK3β by controlling the opposed activities of Akt and calcineurin on this target. Regarding the possible role of PKC in mania, in “manic” BStac mice PKC inhibition reduced amphetamine-induced hyperactivity in the activity cage but barely diminished their swim activity in the FST. It is worth noting that inhibition of PKC increased P-S9 GSK3β but did not diminish the activating P-Y216 form, which clearly prevailed in BStac mice. Thus, PKC just controls the activity of a limited pool of GSK3β^29^^, and present study,^ enough to dampen the effects of psychostimulants but not the effects of more stressful paradigms, such as the FST. In BStac mice, high P-Y216 GSK3β levels prevented PKC inhibition from reducing their manic-like behavior via increases of P-S9 GSK3β. In HINT1^−/−^ mice, GSK3β regulatory phosphorylation levels were moderate and below those seen in HINT1^+/+^ WT mice. This observation raises the question of whether low P-Y216 GSK3β levels such as those observed in HINT1^−/−^ mice can promote manic-like behaviors. Notably, in HINT1-defficient mice PKC inhibition reduced amphetamine-induced hyperactivity and increased their immobility period in the FST. It is worth noting that HINT1^+/+^ WT, BScr or BStac mice did not exhibit increased NR2B/NR2A ratios, and in these mice PKC inhibition did not change or moderately changed their behavior during the FST. Thus, our data suggest that in HINT1^−/−^ mice the PKC-regulated pool of GSK3β collaborate with NR2B-enriched NMDARs to facilitate synaptic LTD, which may explain the simultaneous involvement of GSK3β and PKC in bipolar mania. In fact, PKC and Akt/GSK3β pathway have been implicated in environment-driven changes in the mood of BPD patients^76^, and our data on PKC and NR2B-enriched NMDARs certainly support the participation of this pathway in these processes. Therefore, enhanced PKC activity in combination with an anomalous NMDAR apparently supports bipolar mania and diminishes stress-mediated recruitment of depressive-like behaviors.

Our study of HINT1^−/−^ mice produced some results that may be relevant to the treatment of human BPD. Our findings regarding the involvement of PKC in BPD indicate that drugs that selectively reduce PKC activity may be therapeutically relevant. The inhibition of PKC reduces pro-manic mechanisms mostly by increasing the availability of NMDAR-dependent depressive behaviors. Indeed, the reported efficacy of tamoxifen in BPD patients^10^ indicates that it may be possible to reduce bipolar mania without triggering excess depressive behaviors. Given that a balance exists between these opposing behaviors, treatments that selectively reduce the pro-manic activity of the mice would indirectly promote reactive depression, but of moderate strength. Valproate and most direct inhibitors of GSK3β exert these effects. We cannot discount the possibility that chronic administration of these drugs may affect PKC activity in addition to exerting the effects described above; however, they may still be of therapeutic interest.

Certain GSK3β inhibitors can also promote antidepressant-like effects^77^,^78^^, and present study^. We observed that 3F8 moderately decreased NMDAR-induced behaviors in HINT1^+/+^ WT mice. However, in mice with increased GSK3β activity, *i.e.,* HINT1^−/−^ and BStac mice, 3F8 barely decreased activity-directed behaviors and recruited NMDAR-mediated depressive-like behaviors. We compared the behavioral effects of 3F8 with those of iPKCs in HINT1^−/−^ mice. iPKCs exhibited a tendency to reduce HINT1^+/+^ WT mice swim activity, but in other mouse strains they clearly exerted antidepressant-like effects^79^. Splicing of the *GSK3β* gene probably accounts for the differences in the ability of GSK3β inhibitors to recruit NMDAR function. A minor (approximately 15% of total) splice variant of GSK3β known as GSK3β2 has a 13-residue insert in its kinase domain and exhibits reduced activity in the presence of several GSK3β substrates. GSK3β2 localizes primarily to neuronal cell bodies, where the majority of NMDARs reside. The un-spliced GSK3β is also present in neuronal processes^80^, including pre-synaptic terminals, where it can control glutamate release. GSK3β inhibitors that can affect the activity of both forms of the enzyme, such as 3F8, could also mimic the beneficial effects of iPKCs in BPD patients.

Thus, the observations from this and previous studies suggest that depression and mania may coexist as opposing forces instead of alternating during BPD. The outcome of this emotional antagonism results in mood swings typical of this mental disorder. The HINT1-knockout mouse is a valid model of human BPD that may be used to discover novel drugs, identify alternative pharmacological targets, and customize treatment protocols dependent on the participation of NMDARs in specific phases of the disease.

## Methods

### Animals

Knock-out mice with the genetic background of 129 mice and exhibiting targeted disruption of the *HINT1* gene (HINT1^−/−^, a gift from I.B. Weinstein/J.B. Wang), WT littermate mice (HINT1^+/+^), and two strains of Black Swiss (BS) mice from the Charles River and Taconic Farms (USA) were used in this study. HINT1^−/−^ mice exhibited no noticeable differences from their HINT1^+/+^ WT littermates with respect to appearance, body size, histologic or morphologic parameters^81^. Genotyping was performed using a previously described protocol^82^, and homozygous transgenic mice were selected and used together with HINT1^+/+^ WT mice in all assays. Male mice were housed at a constant temperature (22 ± 1 °C) under a 12/12 h light-dark cycle and were allowed unlimited access to food and water. To reduce the risk of social stress, mice from the same litter were grouped together remained in these groups throughout the study. The mice were also provided extra space for comfort, as well as nesting material (*e.g*., soft paper and cardboard refuge) and small pieces of chewable wood. Experiments were regularly performed in different cohorts of mice to avoid any variations caused by handling stress or exposure to stressful paradigms. Because stress determines the subsequent behavior of HINT1^−/−^ mice, all the mice were used only once. An exception was made for the mice that were used in experiments designed to study the influence of stress on behavior. These animals were used twice. Molecular determinations were performed in mice that received only the drugs under study and in mice that were also subjected to the corresponding behavioral tests after drugs administration. All experiments were performed according to the European regulations for experimental work with animals (directive 2010/63/EU) and were approved by the Ethics Committee for Animal Research of CSIC. Behavioral test procedures are provided in the Supplementary Information.

### Membrane preparation and protein detection

These procedures used for these assays have been described extensively elsewhere^22^,^83^. Briefly, frontal cortices were obtained from groups of 6 to 10 mice that were euthanized via decapitation. Proteins from solubilized synaptosomes were resolved by SDS/polyacrylamide gel electrophoresis (PAGE), transferred onto 0.2 μm polyvinylidene difluoride (PVDF) membranes (#162–0176, Bio-Rad, Madrid, Spain), probed overnight at 6 °C with the appropriate primary antibodies (see Supplementary Information) diluted in Tris buffered saline (pH 7.7) + 0.05% Tween-20, and then detected with the appropriate secondary antibodies (2 h) conjugated to horseradish peroxidase. Antibody binding was visualized via chemiluminescence (#170-5061, Bio-Rad, Madrid, Spain) and was recorded with a ChemiImager IS-5500 (Alpha Innotech, San Leandro, California). Densitometry was performed using Quantity One software (Bio-Rad), and the results are expressed as the mean of the integrated volume (average optical density of the pixels within the object area/mm^2^).

### Enzymatic activity

Pep tag protein kinase C and protein kinase A assays (Promega V5330 and V5340) were performed to assess total kinase enzymatic activity. The supernatants from cortical brain lysates were incubated with specific substrates. The peptides that served as substrate were separated according to their net charges via electrophoresis on a 0.8% agarose gel in 50 mM Tris–HCl (pH 8.0). A colorimetric assay kit (Enzo, BML-AK816-0001) was used to measure cellular calcineurin (PP2B) phosphatase activity, according to the manufacturer’s instructions.

### Permanent focal cerebral ischemia (pMCAO) and determination of infarct size

Focal cerebral ischemia was induced via permanent occlusion of the middle cerebral artery (pMCAO), as previously described^22^. Briefly, a small craniotomy incision was made over the trunk of the right middle cerebral artery (MCA) and above the rhinal fissure. After its identification, the dura mater was resected, and the MCA was ligated just before the bifurcation of its frontal and parietal branches using 9-0 suture. Sham-operated animals were subjected to an identical procedure, although their MCAs were not ligated. Following surgery, the mice were returned to their cages, where they were maintained at room temperature and allowed free access to food and water. Complete interruption of blood flow was confirmed via an operating microscope. For infarct size determination at 48 hours after MCAO, magnetic resonance imaging was performed using a BIOSPEC BMT 47/40 (Bruker, Ettlingen, Germany), and infarct volumes were calculated from T2-weighted images using ImageJ 1.44l software (NIH, Bethesda, MD, USA).

### Artwork and statistical analysis

All graphs and statistical analyses were generated and performed using the Sigmaplot/SigmaStat v.13 package (SPSS Science Software, Erkrath, Germany). Significance was defined as *p* < 0.05. Data were analyzed using paired t test, or one-way ANOVA followed by all pairwise Holm-Sidak multiple comparison tests or Dunnett multiple comparisons vs the control group, as indicated in the legends to the Figures.

## Additional Information

**How to cite this article:** Garzón-Niño, J. *et al*. Increased PKC activity and altered GSK3β/NMDAR function drive behavior cycling in HINT1-deficient mice: bipolarity or opposing forces. *Sci. Rep.* **7**, 43468; doi: 10.1038/srep43468 (2017).

**Publisher's note:** Springer Nature remains neutral with regard to jurisdictional claims in published maps and institutional affiliations.

## Supplementary Material

Supplementary Information
