# Supplementary material for: Increased PKC activity and altered GSK3β/NMDAR function drive behavior cycling in HINT1-deficient mice: bipolarity or opposing forces
Source: Sci Rep. 2017 Feb 27;7:43468. doi: 10.1038/srep43468 (PMC5327482; doi:10.1038/srep43468)
Supplement: Supplementary Information [file srep43468-s1.pdf]

Increased PKC activity and altered GSK3β/NMDAR function in manic-depressive HINT1-deficient mice: bipolarity or opposing forces

Javier Garzón*, María Rodríguez-Muñoz, Elsa Cortés-Montero, and Pilar Sánchez-Blázquez

Supplementary Materials and Methods

Behavioral tests

Before beginning behavioral testing, we allowed the mice to familiarize themselves with the testing room for two consecutive days (60 min/day). On the day of testing, we transferred the mice to the testing room 30 min prior to each test session. To exclude the effects of potential changes in behavior, we performed each test with a different cohort of animals. The BStac mice sub-strain was included in some experiments, as those mice have been considered useful for modeling some of the domains of mania (1,2). In addition, a normal BS sub-strain was included in the study to serve as a WT reference for BStac mice.

Spontaneous activity. The mice were tested individually for spontaneous activity using 20 × 20 × 28 cm transparent plastic automated activity monitors (Accuscan activity analyzer - Versamax 260 v2.4; Omnitech Electronics, Inc., OH, USA). Infrared beam crossings were recorded for 100 min at 10 min intervals. At the end of each session, the mice were returned to their home cages, and the boxes were wiped clean with a 10% alcohol solution.

Response to psychostimulants. We tested separate cohorts of $HINT1^{+/+}$ WT, $HINT1^{-/-}$, and BStac mice to evaluate the hyperlocomotor-inducing effects of psychostimulants. The mice were allowed a 30 min period of habituation and were then injected with apomorphine (5 mg/kg, ip) or amphetamine (2 mg/kg, ip) and returned to their activity cages. Additionally, the horizontal activities of mice pre-treated with the selective GSK3β inhibitor TDZD8 (30 mg/kg, ip), iPKC Gö7874 (1 nmol, icv), or mild acute stressor were recorded during 30 min of spontaneous activity and for 70 min following vehicle (saline) challenge.

Sweet solution preference test. Over a period of 48 h, the mice were given the option of consuming a 1% sucrose solution (SIGMA, Madrid, Spain) in addition to their regular supply of water and food. The sucrose concentration was selected based on previous study involving

BS mice (1). The sweet solution bottle was made available to the mice throughout the entire preference test period. Sucrose solution and water bottle weights were recorded at the beginning of the experiment and every 24 h thereafter. To avoid the possible effect of side preference on drinking behavior, we changed the positions of the bottles every 12 h. The mice were not subjected to food or water deprivation prior to the test. Preference for sucrose was calculated as the percentage of sucrose solution consumed relative to the total amount of liquid consumed. Preference was measured over 2 days because it was previously demonstrated that differences between groups appeared within 2 days (1).

Resident-intruder test. This test was used to evaluate the aggression element of manic behavior (2). Resident animals were singly housed, and no cage changes were performed during the week prior to testing. The mice were transferred from their home cages to the experimental room, where their cage covers were removed. After 5 min of adaptation period, a younger strain-matched, previously group-housed mouse (intruder) was placed in the home cage of each singly housed resident animal. Resident behavior was recorded during a 10 min session. Each intruder mouse was used only once. Non-interaction behaviors were not scored. Each resident animal aggression score was calculated as the ratio of aggressive interactions to total (aggressive + non-aggressive) interactions. To minimize animal harm, the mice were separated briefly following attacks. At the end of the session, the intruder was removed, and both mice were returned to the colony room.

Forced swim test (FST). This test was based on the original version of the Porsolt forced swim test for mice (3). Mice were placed in a vertical cylindrical container (25 cm tall × 20 cm diameter), filled with tap water at 22 ± 1 °C. Thus, the mice swam without the possibility of touching the bottom. The mice were placed in the water during 6 min and their activity and immobility periods were recorded during the last 4 min. At the end of the session, the mice

were removed from the water, dried with a paper towel, and placed back in their home cages. The water in the container was changed after each session. Scored behaviors were defined as active (swim and struggle) versus passive (floating with only minimal movements needed to keep head above water) behaviors.

Tail suspension test. Mice were individually suspended by the tail from a hook raised 20 cm above the floor using adhesive tape placed 2 cm from the tip of the tail. Test sessions lasted for 6 min and were videotaped and subsequently scored by a trained observer. The following behaviors were rated: (1) immobility – mice were considered immobile when they hung by their tails without engaging in any active behaviors; (2) swinging – mice were considered to be swinging when they continuously moved their paws vertically while keeping their bodies straight and/or moved their bodies from side to side; and (3) curling – mice were considered to be curling when they engaged in active twisting movements involving their entire bodies.

Passive avoidance task. Acquisition and retention of passive avoidance behaviors were examined using identical illuminated and non-illuminated ($20 \times 10 \times 15$ cm^3) boxes separated by a guillotine door (5×5 cm^2), as described elsewhere (4). Mice participated in two separate trials. First, in the acquisition trial, each mouse was initially placed in the light compartment, and the door between the two compartments was opened after 10 s. When the mouse entered the dark compartment, the guillotine door automatically closed, and an electrical foot shock (0.5 mA, 3 s) was delivered through the floor. The latency time before crossing into the dark chamber was recorded. Only mice that entered the dark chamber within 60 s were subjected to a retention trial. For the retention trial, each mouse was again placed in the light compartment, and the latency time before crossing into the dark compartment was recorded (up to 10 min).

Drugs and drug administration.

(+)-MK801 maleate, D-AP5, (R)-(-)-apomorphine hydrochloride, D-amphetamine sulfate, TCG24, 3F8, lithium carbonate, citalopram hydrobromide, bupropion hydrochloride, amitriptyline hydrochloride, chelerythrine chloride, risperidone, and lamotrigine were obtained from Tocris (UK). Valproic acid sodium salt, TDZD8, and imipramine hydrochloride were obtained from Sigma-Aldrich (Spain). Gö7874, Bisindolylmaleimide 1 hydrochloride and the PKA inhibitor 14-22 amide were purchased from Merck-Millipore (Spain). Drug doses were chosen based on their effects in animal models of affective disorders, dissolved in ethanol/Cremophor EL/physiological saline (1:1:18), and administered via intraperitoneal (ip) injections at a dose of 10 mL/kg body weight. Vehicle control animals received equivalent volumes of saline. In some experiments, the compounds were intracerebroventricularly (icv) administered, as described elsewhere (5).

Antibodies

The following primary antibodies were used in this study: anti-GSK3β (#9315 Cell Signaling), anti-GAP43 (#AB5220 Millipore), anti-NR1 (#MAB1586 Millipore), anti-NR1C1 (#AB5046P Millipore), anti-NR2A (#ab14596 Abcam), anti-NR2B (#ab14400 Abcam), anti-Akt (#4691 Cell Signaling Technology), anti-β-catenin (#9582 Cell Signaling Technology), anti-P-S9 GSK3β (#9336 Cell Signaling Technology), anti-P-Y216 GSK3β (#ab75745 Abcam), anti-P-S41 GAP43 (#AB5401 Millipore), anti-P-S473 Akt (#4060 Cell Signaling Technology), anti-P-T308 Akt (#2965 Cell Signaling Technology), anti-P-S33/37, P-T41 β-catenin (#9561 Cell Signaling Technology), anti-protein phosphatase 1 (PP1) (sc-443 SCBT), anti-protein phosphatase inhibitor 1 (ab40877), anti-protein phosphatase 1 inhibitor subunit 2 (phospho T72) (ab 27850), and anti-αtubulin (#T9026 Sigma).

Adult hippocampal cell proliferation

For immunohistochemical analysis of cell proliferation, mice received BrdU (4 × 75 mg/kg

every 2 h, ip; Sigma) in sterile 0.9% NaCl solution on the last day of antidepressant treatment and 24 h before euthanasia. For quantification of BrdU-positive cells every sixth section corresponding to interaural stereotaxic coordinates ranging from 4.48 to 5.70 mm throughout the hippocampus was processed and counted under a light microscope (Carl Zeiss Axioskop 2 Plus) at 40× and 100× magnification. The total numbers of BrdU-positive cells per section were determined and multiplied by 6 to obtain the total number of BrdU-positive cells per hippocampus.

Hippocampal slice preparation and electrophysiology

Cortical hippocampal slices were obtained from wild type HINT1$^{+/+}$ WT and HINT1$^{-/-}$ mice (13–15 days old). The animals were decapitated, and their brains were rapidly removed and placed in ice-cold artificial cerebrospinal fluid containing (in mM) 124 NaCl, 2.7 KCl, 1.25 KH_2PO_4, 2 $MgSO_4$, 26 $NaHCO_3$, 10 glucose, 0.4 ascorbic acid, and 2 $CaCl_2$ and gassed with 95% O_2/5% CO_2 (pH 7.3). Slices were incubated for 45 minutes at room temperature and then transferred to an immersion recording chamber and superfused with Mg^{2+}-free ice-cold artificial cerebrospinal fluid (ACSF) containing (in mM) 124 NaCl, 2.7 KCl, 1.25 KH_2PO_4, 26 $NaHCO_3$, 10 glucose, 4 $CaCl_2$, 0.01 glycine, and 0.05 picrotoxin (to block GABA A receptors) and gassed with 95% O_2/5% CO_2 (pH 7.3). Extracellular Mg^{2+} was omitted to maximize NMDAR-mediated currents. Cells were visualized under an Olympus BX50WI microscope equipped with infrared and differential interference contrast imaging devices and a 40x water-immersion objective. Electrophysiological recordings from CA1 pyramidal neurons were performed using the whole-cell patch-clamp technique with an internal solution containing (in mM) 135 potassium gluconate, 10 KCl, 10 HEPES, 1 $MgCl_2$ and 2 ATP-Na_2 (pH 7.4). Neuron activity was recorded under voltage-clamp conditions with the membrane potential maintained at -70 mV. NMDA receptor-mediated currents were isolated in the

presence of 6-cyano-7-nitroquinoxaline-2,3-dione (CNQX; to block AMPA-kainate receptors; 20 μM). When indicated, purified rHINT1 protein was included in the internal solution at a final concentration of 150 nM. The effects of the NMDAR antagonist D-AP5 (50 μM) and the iPKC bisindolylmaleimide I (Bis1, 1 μM) were evaluated. Signals were fed to a Pentium-based PC through a DigiData 1440A interface board. Signals were filtered at a 1 kHz rate and acquired at a 10 kHz sampling rate. pCLAMP 10.2 (Axon instruments, Sunnyvale, CA) software was used for data acquisition and storage.

References

1. Flaisher-Grinberg,S., Overgaard,S., & Einat,H. Attenuation of high sweet solution preference by mood stabilizers: a possible mouse model for the increased reward-seeking domain of mania. *J Neurosci. Methods* **177**, 44-50 (2009).

2. Einat,H. Establishment of a battery of simple models for facets of bipolar disorder: a practical approach to achieve increased validity, better screening and possible insights into endophenotypes of disease. *Behav. Genet.* **37**, 244-255 (2007).

3. Porsolt,R.D., Bertin,A., & Jalfre,M. Behavioral despair in mice: a primary screening test for antidepressants. *Arch. Int. Pharmacodyn. Ther.* **229**, 327-336 (1977).

4. Kim,D.H. *et al.* The effects of acute and repeated oroxylin A treatments on Abeta(25-35)-induced memory impairment in mice. *Neuropharmacology* **55**, 639-647 (2008).

5. Rodríguez-Muñoz,M., Sánchez-Blázquez,P., Vicente-Sánchez,A., Berrocoso,E., & Garzón,J. The mu-opioid receptor and the NMDA receptor associate in PAG neurons: implications in pain control. *Neuropsychopharmacology* **37**, 338-349 (2012).

Supplementary Figures

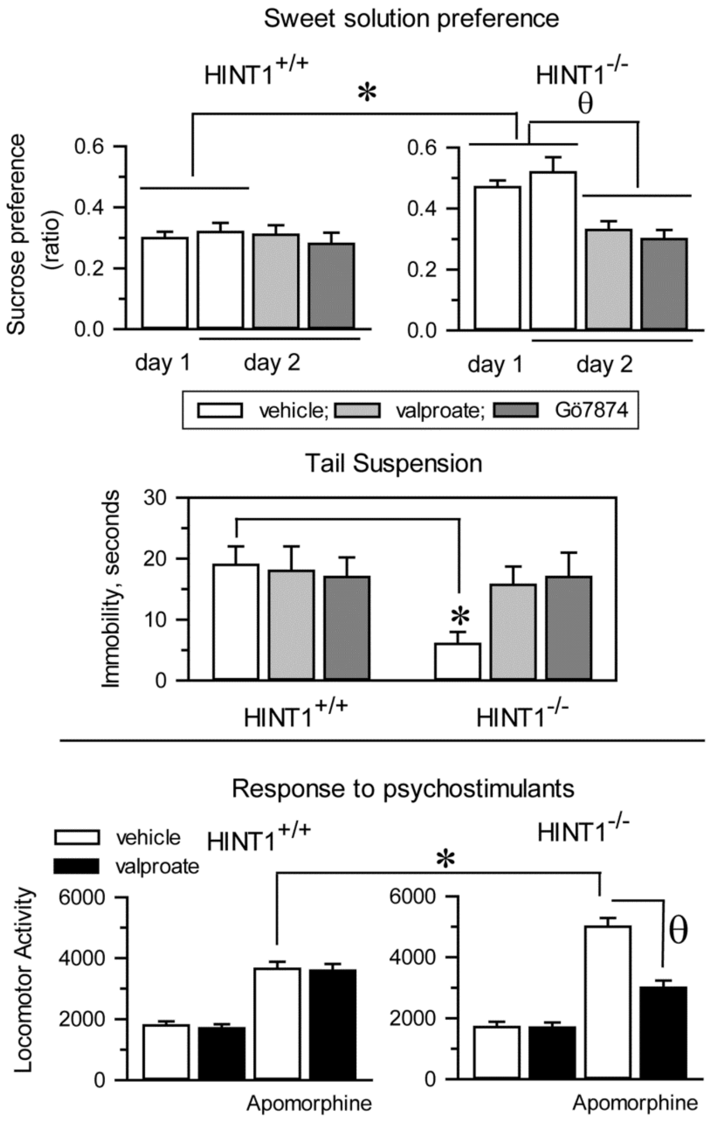

Fig. S1. Mood stabilizers, such as valproate, and PKC inhibitors diminish manic-like behaviors. In HINT1$^{-/-}$ mice, but not in WT mice, valproate (200 mg/kg, ip) and Gö7874 (1 nmol, icv) reduced the preference for saccharin and hypersensitivity to apomorphine (5 mg/kg, ip) and increased immobility scores during the TST. The results are expressed as the mean ± SEM of total scores (n=6/group). *Significant difference between the groups receiving vehicle instead of treatment; θSignificant effect of treatments compared to vehicle. ANOVA, Dunnett multiple comparisons vs control group, p<0.05.

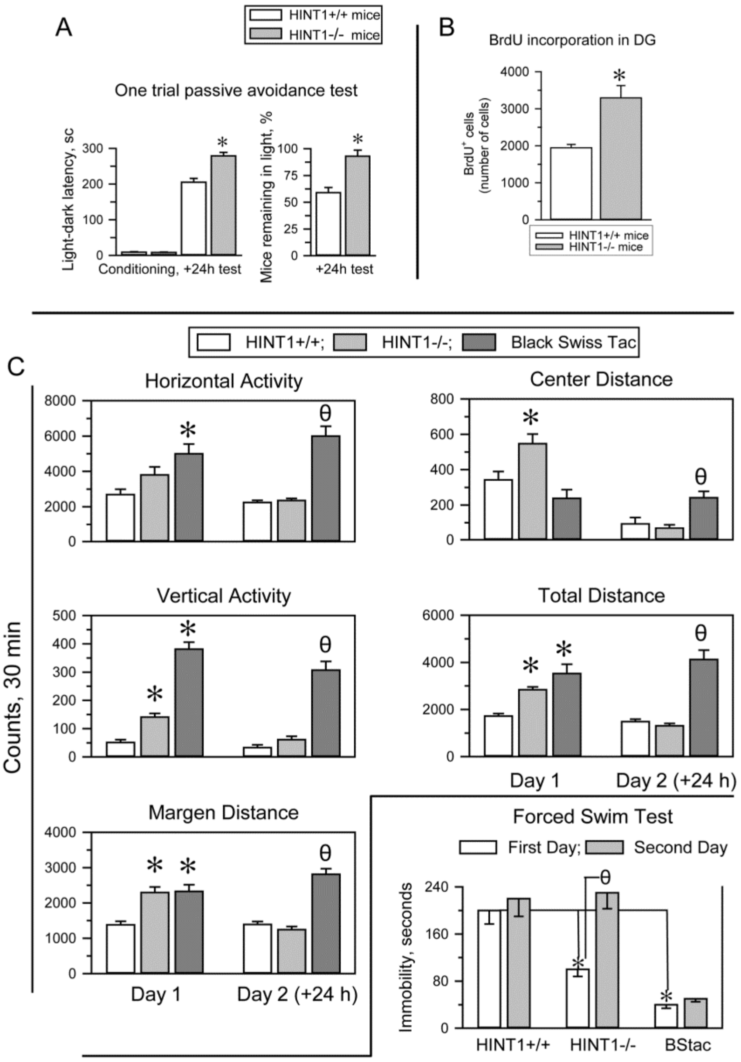

Fig. S2. Differential effects of stress on behaviors exhibited by WT, HINT1$^{-/-}$, and BStac mice. (**A**) Performances of HINT1$^{+/+}$ and HINT1$^{-/-}$ mice on the passive avoidance test. (**B**) Proliferating cells in the dentate gyrus (DG) of the hippocampus of HINT1$^{+/+}$ and HINT1$^{-/-}$ mice. *Significantly different, paired *t*-test, $p<0.05$. (**C**) Comparative study of HINT1$^{+/+}$, HINT1$^{-/-}$, and BStac mice performances in activity cages and on the FST. The assay was conducted for two consecutive days. Each bar is the computed mean ± SEM of individual scores (n=10/group). *Significantly different from the HINT1$^{+/+}$ mice data, day 1; θ, day 2 (+ 24 h). ANOVA, Dunnett multiple comparisons vs control group, $p<0.05$.

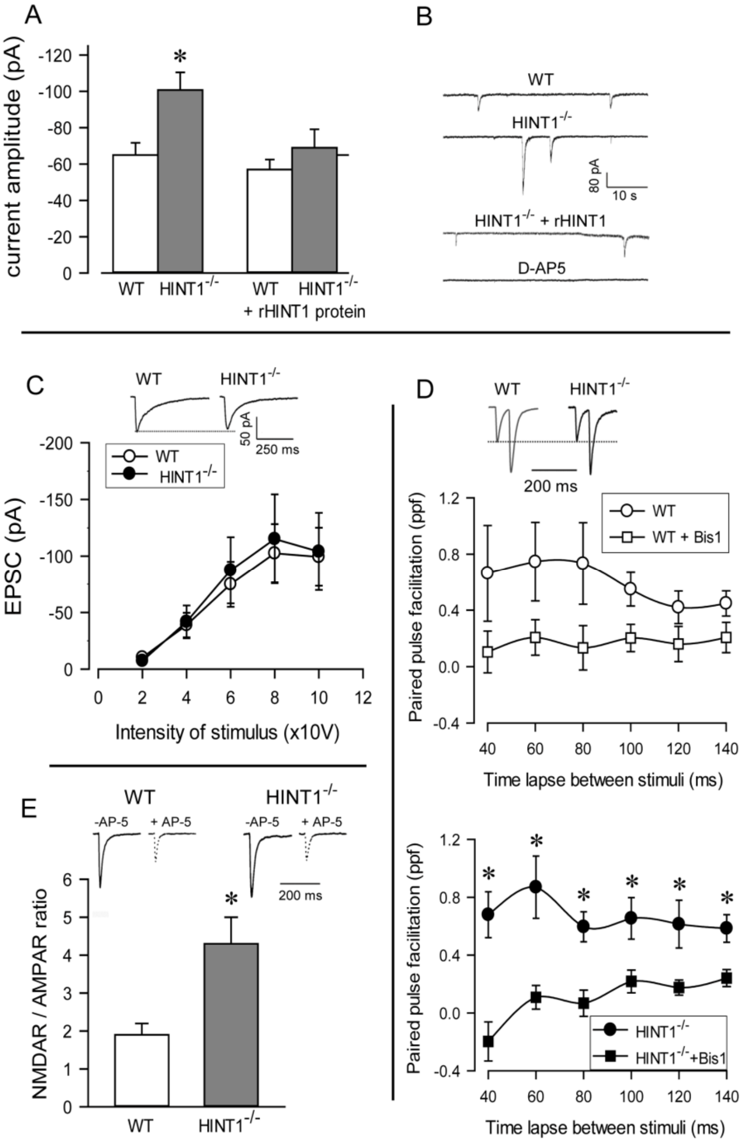

Fig. S3. Spontaneous NMDAR-mediated currents in CA1 pyramidal neurons from WT and HINT1$^{-/-}$ mice. (**A**) The mean amplitudes of the spontaneous, NMDAR-mediated currents recorded in neurons from WT and HINT1$^{-/-}$ mice under controlled conditions and when recombinant rHINT1 was included in the solution of the recording pipette. The data are represented as the mean ± SEM of the WT (n=12) and HINT1$^{-/-}$ (n=28) determinations. (**B**) Representative whole-cell currents recorded from CA1 pyramidal neurons from WT and HINT1$^{-/-}$ mice. Higher NMDAR-mediated slow inward current amplitudes were observed in HINT1$^{-/-}$ mice than in WT mice, and these amplitudes normalized in the presence of rHINT1. The NMDAR antagonist D-AP5 (50 μM) abolished these currents. (**C**) Synaptic transmission properties were unaffected in HINT1$^{-/-}$ mice. Top, representative NMDA receptor-mediated excitatory postsynaptic currents (EPSCs) evoked by Schaffer collateral (SC) stimulation recorded in the CA1 pyramidal neurons of WT (left) and HINT1$^{-/-}$ (right) mice. Bottom, mean amplitudes of the isolated NMDA components of the

EPSCs evoked at different intensities of SC stimulation in structures from wild-type (n=8) and HINT1$^{-/-}$ (n=14) mice. (**D**) Top, representative paired-pulse EPSCs from WT (left) and HINT1$^{-/-}$ (right) mice. Bottom, paired-pulse facilitation (ppf) at different time intervals between stimuli in wild-type (n=15) and HINT1$^{-/-}$ (n=10) mice in the presence and absence of a PKC inhibitor (Bis1). (**E**) Representative EPSCs in the absence and presence of D-AP5 (to isolate the NMDA receptor component) in wild-type (left) and HINT1$^{-/-}$ (right) mice. Bottom, NMDA/AMPA ratio of the EPSCs in wild-type (n=6) and HINT1$^{-/-}$ (n=9) mice. The data are represented as the mean ± SEM. *Significantly different. ANOVA, Dunnett multiple comparisons vs control group, $p<0.05$.

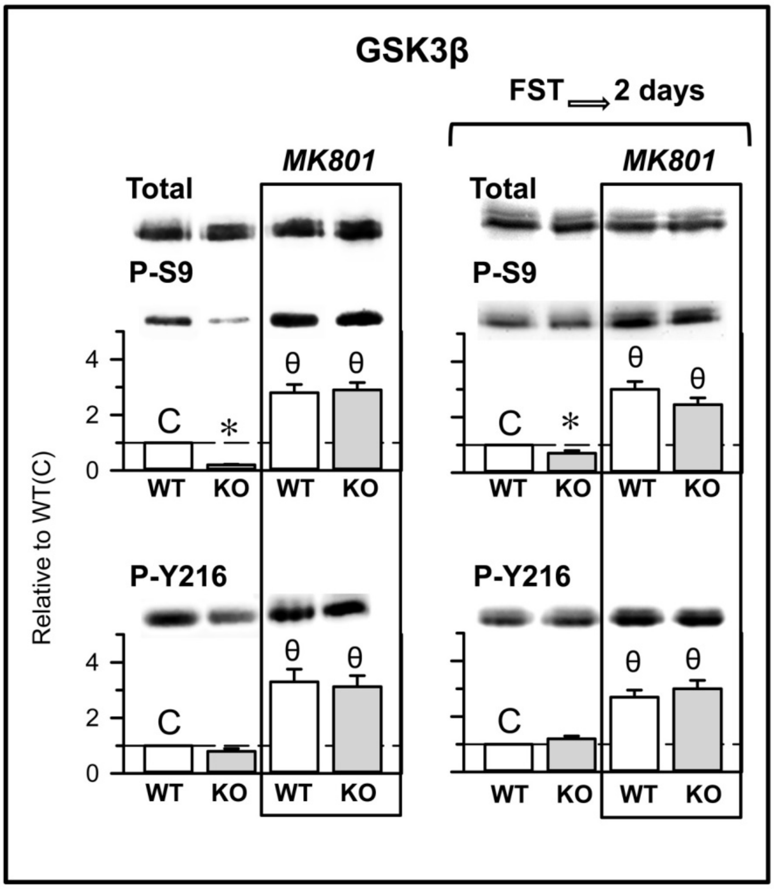

Fig. S4. Effect of MK801 administration on GSK3β content and phosphorylation in the frontal cortices of HINT1$^{+/+}$ and HINT1$^{-/-}$ mice. (**A**) Mice were treated with saline or MK801 (1nmol, icv). The frontal cortices of groups of 3 mice were removed and pooled for ex vivo molecular analysis. The bars are the mean ± SEM (Sigmaplot v13) of three assays performed on samples obtained from independent groups of mice. Representative blots are shown. Immunosignals (average optical density of the pixels within the object area/mm^2, Quantity One Software; Bio-Rad, Madrid, Spain) were expressed as changes relative to the WT control group (C, attributed an arbitrary value of 1). Tubulin was used as a loading control. *Significant difference from the WT group; θ indicates the corresponding group not receiving MK801. ANOVA, Dunnett multiple comparisons vs control group, *p*<0.05.
